# Development, validation and recalibration of a prediction model for prediabetes: an EHR and NHANES-based study

**DOI:** 10.1186/s12911-024-02803-w

**Published:** 2024-12-18

**Authors:** Nicholas J. Casacchia, Kristin M. Lenoir, Joseph Rigdon, Brian J. Wells

**Affiliations:** 1 Center for Value-Based Care Research, Primary Care Institute, Cleveland Clinic, 9500 Euclid Ave, G10, Cleveland, OH 44195 USA; 2 Division of Public Health Sciences, Department of Biostatistics and Data Science, Wake Forest University School of Medicine, 525 Vine St, Winston-Salem, NC 27101 USA

**Keywords:** Electronic health records, NHANES, Prediabetes, Prediction model, Logistic regression, LASSO, Calibration, External validation

## Abstract

**Background:**

A prediction model that estimates the risk of elevated glycated hemoglobin (HbA1c) was developed from electronic health record (EHR) data to identify adult patients at risk for prediabetes who may otherwise go undetected. We aimed to assess the internal performance of a new penalized regression model using the same EHR data and compare it to the previously developed stepdown approximation for predicting HbA1c ≥ 5.7%, the cut-off for prediabetes. Additionally, we sought to externally validate and recalibrate the approximation model using 2017–2020 pre-pandemic National Health and Nutrition Examination Survey (NHANES) data.

**Methods:**

We developed logistic regression models using EHR data through two approaches: the Least Absolute Shrinkage and Selection Operator (LASSO) and stepdown approximation. Internal validation was performed using the bootstrap method, with internal performance evaluated by the Brier score, C-statistic, calibration intercept and slope, and the integrated calibration index. We externally validated the approximation model by applying original model coefficients to NHANES, and we examined the approximation model’s performance after recalibration in NHANES.

**Results:**

The EHR cohort included 22,635 patients, with 26% identified as having prediabetes. Both the LASSO and approximation models demonstrated similar discrimination in the EHR cohort, with optimism-corrected C-statistics of 0.760 and 0.763, respectively. The LASSO model included 23 predictor variables, while the approximation model contained 8. Among the 2,348 NHANES participants who met the inclusion criteria, 30.1% had prediabetes. External validation of the LASSO model was not possible due to the unavailability of some predictor variables. The approximation model discriminated well in the NHANES dataset, achieving a C-statistic of 0.787.

**Conclusion:**

The approximation method demonstrated comparable performance to LASSO in the EHR development cohort, making it a viable option for healthcare organizations with limited resources to collect a comprehensive set of candidate predictor variables. NHANES data may be suitable for externally validating a clinical prediction model developed with EHR data to assess generalizability to a nationally representative sample, depending on the model’s intended use and the alignment of predictor variable definitions with those used in the model’s original development.

**Supplementary Information:**

The online version contains supplementary material available at 10.1186/s12911-024-02803-w.

## Background

Over 1 in 3 American adults have prediabetes [1], defined as having an elevated glycated hemoglobin (HbA1c) ≥ 5.7%, and 80% are unaware that they have it [1]. Additionally, adults with undiagnosed diabetes comprise 23.0% of prevalent diabetes cases in the United States [2]. Patients who go on to develop diabetes are at increased risk of cardiovascular complications [3], and have, on average, healthcare expenses that are 2.3 times higher than persons not diagnosed with diabetes [4]. As such, screening patients for early detection of elevated HbA1c facilitates early intervention, which may prevent or delay disease progression, prevent micro- and macrovascular complications, mitigate unnecessary healthcare expenditures attributable to diabetes, and improve patient outcomes [5]. Diagnostic prediction models that estimate the risk of elevated HbA1c can be implemented into the electronic health record (EHR) to identify those at risk for prediabetes who may otherwise go undetected.

The American Diabetes Association (ADA) and Centers for Disease Control and Prevention (CDC) offer a prediabetes risk test that includes history of gestational diabetes if female, family history of diabetes, physical activity, history of hypertension, patient age, sex, height, and weight [6]. The CDC Risk Score model, originally developed with NHANES 1999–2004 and validated with NHANES 2005–2006 [7], demonstrated poor performance in NHANES 2013–2014 [8]. This decline may be due to differences in predictor variable definitions and temporal changes in prediabetes prevalence. Despite it hovering just above the conventional level of significance, physical inactivity was included in the CDC model because of its protective and modifiable nature [7]. However, variables collected in health surveys, such as physical activity, are not always routinely captured in EHRs, complicating the application of survey-developed models to clinical settings.

Previously, Wells et al. [9] built a logistic regression model from EHR data using stepdown approximation to predict prediabetes. The model-development cohort comprised adult patients of Atrium Health Wake Forest Baptist Medical Center (AHWFBMC) in Winston-Salem, North Carolina who had undergone HbA1c testing, had prior evidence of hyperglycemia, or had a prescription for an antihyperglycemic medication between September 2012 and September 2016 [9]. The selection of candidate predictors was guided by their theoretical relationship to hyperglycemia [9]. Harrell’s model approximation method was used to derive the most parsimonious model [10]. The following predictors were selected from a larger subset of candidate variables in order from most to least importance: age, body mass index (BMI), random glucose, race, serum non–high-density lipoprotein (non-HDL), serum total cholesterol, estimated glomerular filtration rate (eGFR), and smoking status [9]. The approximation model was internally validated using tenfold cross-validation and outperformed alternative models with a C-statistic of 0.765 [9].

Alhassan et al. [11] replicated the Wells et al. approximation model using EHR data from Saudi Arabia by building three models using identical predictors with the exception of race, which was uniform across their patient population, and smoking status, which was absent from their dataset. While the omission of race and smoking status limited the external validation of the original model, they replicated the logistic regression equation used in the original model and validated it through tenfold cross-validation, which yielded commendable accuracy and calibration [11]. Alhassan et al. found that the model with fewer predictors performed the best and that the order of variable importance (most to least important: random glucose, age, eGFR, cholesterol, non-HDL, and BMI) differed from Wells et al. [11] Systematic population differences (e.g., location, data collection processes, and individual characteristics) likely contributed to dissimilarities in variable importance.

Generally, clinical prediction models perform better on the dataset used for development than on new patient populations [12, 13]. External validation is essential for evaluating a model’s predictive performance on a separate dataset that was not part of the model’s development [14, 15]. Assessing a prediction model's performance on new data is crucial for testing its generalizability and transportability, ensuring that it can reliably support decision-making in new patient populations before widespread implementation [12–15]. Although the Wells et al. model was replicated, it has not been compared to a model using a different variable selection technique within the same development cohort. First, we aimed to compare the performance of the prediabetes diagnostic model developed using the original approximation stepdown procedure to the LASSO method, as these two approaches offer distinct advantages: the approximation method focuses on creating a parsimonious model by retaining the most significant predictors and may be easier to interpret, while LASSO shrinks less relevant predictors toward zero, potentially enhancing model simplicity and performance [10, 16]. By comparing these two methods, we sought to ensure the robustness and potential advantages of each approach in improving predictive performance. Additionally, we aimed to externally validate and recalibrate the approximation model using NHANES data to assess its generalizability and transportability.

## Methods

The Wake Forest University Health Sciences institutional review board approved this study (IRB00031798) and waived informed consent. This study conformed to the Transparent Reporting of a multivariable prediction model for Individual Prognosis Or Diagnosis (TRIPOD + AI) checklist [17]. The development cohort was identical to that used in Wells et al. [9], and 35 candidate predictor variables were considered (sirolimus was not considered due to low prevalence). The outcome of interest was defined as HbA1c ≥ 5.7%, or prediabetes, per the original study [9] and the ADA’s diagnostic criteria [18]. Given that we used the same dataset previously utilized by Wells et al. for model development, we verified the appropriateness of the sample size using the “pmsampsize” package [19, 20]. Based on an outcome prevalence of 26% [9], a C-statistic of 0.76 [9], and 35 candidate predictors, we calculated that a minimum sample size of 1,789 with 446 events was required for model development that ensured a shrinkage factor of ≥ 0.9, an absolute difference of ≤ 0.05 between the model's apparent and adjusted proportion of variance explained, and a margin of error ≤ 0.05 in the estimate of average outcome risk. LASSO and approximation logistic regression models were built to predict the probability of prediabetes. For the LASSO logistic regression, tenfold cross-validation was used to select the largest lambda at which the deviance was within one standard error of the minimal deviance. For the stepdown approximation procedure, we first fit a full logistic regression model in which continuous variables were fit using the restricted cubic splines function with 3 knots. Then, an ordinary least squares model was used to approximate the linear predictor of the full model. Variables were removed using backward elimination until the R-squared value reached 0.95 [10]. Both the LASSO and approximation models were internally validated using 2,000 bootstrap resamples whereby the entire modeling process (including tuning parameter selection via tenfold cross-validation for the LASSO model and backward elimination for the approximation model) was repeated for each resample to obtain an optimism-corrected C-statistic, calibration intercept, calibration slope, Brier score, integrated calibration index (ICI) [21, 22], and a bias-corrected calibration curve. The stability of the LASSO and approximation models was also assessed by performing 2,000 bootstrap resamples, after which we (1) calculated the average mean absolute difference between individuals' original predictions and those from the bootstrap models, and (2) generated mean absolute predictor error plots, prediction instability plots, and calibration instability plots for the two models [23].

The 2017–2020 pre-pandemic cycle of NHANES was used for external validation [24]. NHANES is a yearly survey of a nationally representative sample, consisting of interviews and physical examinations, designed to evaluate the health of adults and children in the United States. Validating the approximation model in NHANES data allowed us to assess (1) its transportability to a population with a different case-mix than the development data, and (2) its generalizability to a nationally representative sample. We selected data necessary to derive the predictor variables that corresponded to the original set of candidate variables used to build the original model [9]. Due to the limited data availability in NHANES, however, we were not able to identify peripheral vascular disease and neuropathy.

We included adult participants (≥ 18 years of age) with an HbA1c. To focus on those with prediabetes who may have been missed and would likely benefit from identification, we excluded participants who indicated that a doctor told them they had prediabetes or diabetes and those who took a medication indicated for diabetes management. Additional eligibility criteria included an indication of fasting status as this conferred that they were more likely to undergo the panel of laboratory tests necessary to derive many of the candidate variables, which was slightly different from the development data definition since fasting status was not reliably documented in the AHWFBMC EHR. Laboratory values for non-HDL were calculated by subtracting HDL from total cholesterol. A binary variable was created for obesity (BMI ≥ 30). We calculated eGFR using the Chronic Kidney Disease Epidemiology Collaboration (CKD-EPI) Creatinine Equation (2021) based on SCr, age, and sex [25]. We identified participants as taking medications only if the prescription container or pharmacy print-out was observed by the interviewer to limit recall bias and accurately capture prescription data. Only complete cases were used since the investigators felt that imputation would not be appropriate at model deployment [9]. NHANES variable definitions are in Additional File 1. The approximation model was externally validated by applying the original regression coefficients to NHANES and calculating the predicted probability of prediabetes. Additionally, we assessed the approximation model’s performance after recalibrating the intercept and overall calibration slope by fitting a logistic regression model to NHANES with the original approximation model linear predictor as the only covariable [26–28]. Predictive performance of the approximation model in NHANES was measured using discrimination (C-statistic), calibration (calibration intercept and slope, ICI), visually by a calibration curve, and simultaneous discrimination and calibration (Brier score). NHANES fasting subsample weights were used to estimate population totals, and external performance metrics were weighted using normalized fasting subsample weights, which were calculated by dividing the fasting subsample weight by the mean of all fasting subsample weights for the NHANES validation cohort [29]. Due to missingness of many candidate variables in NHANES we were not able to externally validate the LASSO model. We verified the appropriateness of the NHANES sample size for external validation using the “pmvalsampsize” package [30]. Based on the observed distribution of the linear predictor for the approximation model in the NHANES sample (mean -1.145, standard deviation 1.374), and an outcome prevalence of 30.1%, a minimum sample size of 2,204 with 664 events was required to externally validate the approximation model that precisely estimated an outcome event proportion of 1 (confidence interval width of 0.2), calibration slope of 1 (confidence interval width of 0.2), and a C-statistic of 0.79 (confidence interval width of 0.1). Statistical analyses were performed in R version 4.4.1 (R Foundation for Statistical Computing) using the “nephro” [31], “glmnet” [32], “rms” (Regression Modeling Strategies), and “CalibrationCurves” [33] packages.

## Results

**Fig. 1:**
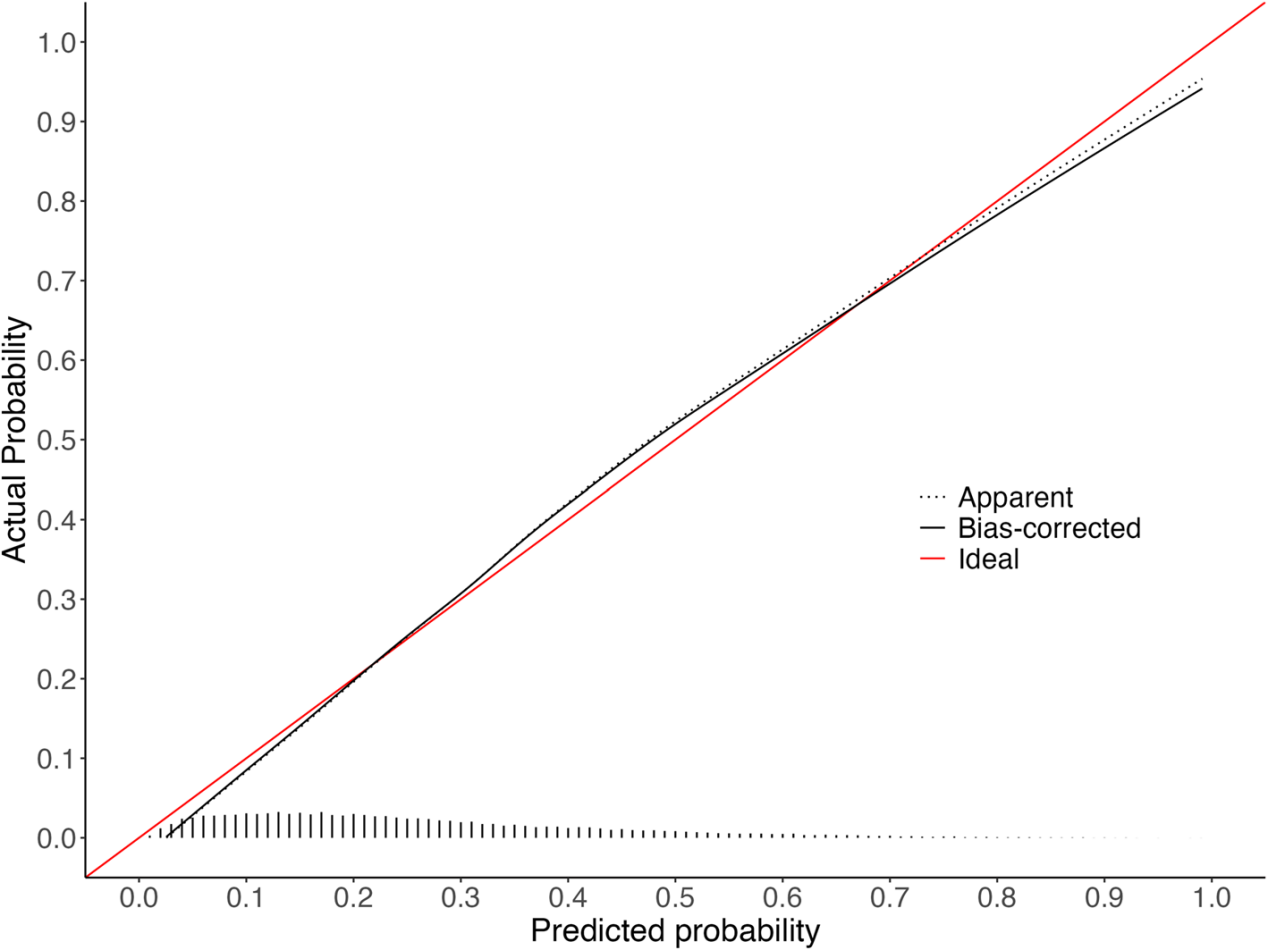
Calibration curve for LASSO model fit on development EHR cohort

**Fig. 2:**
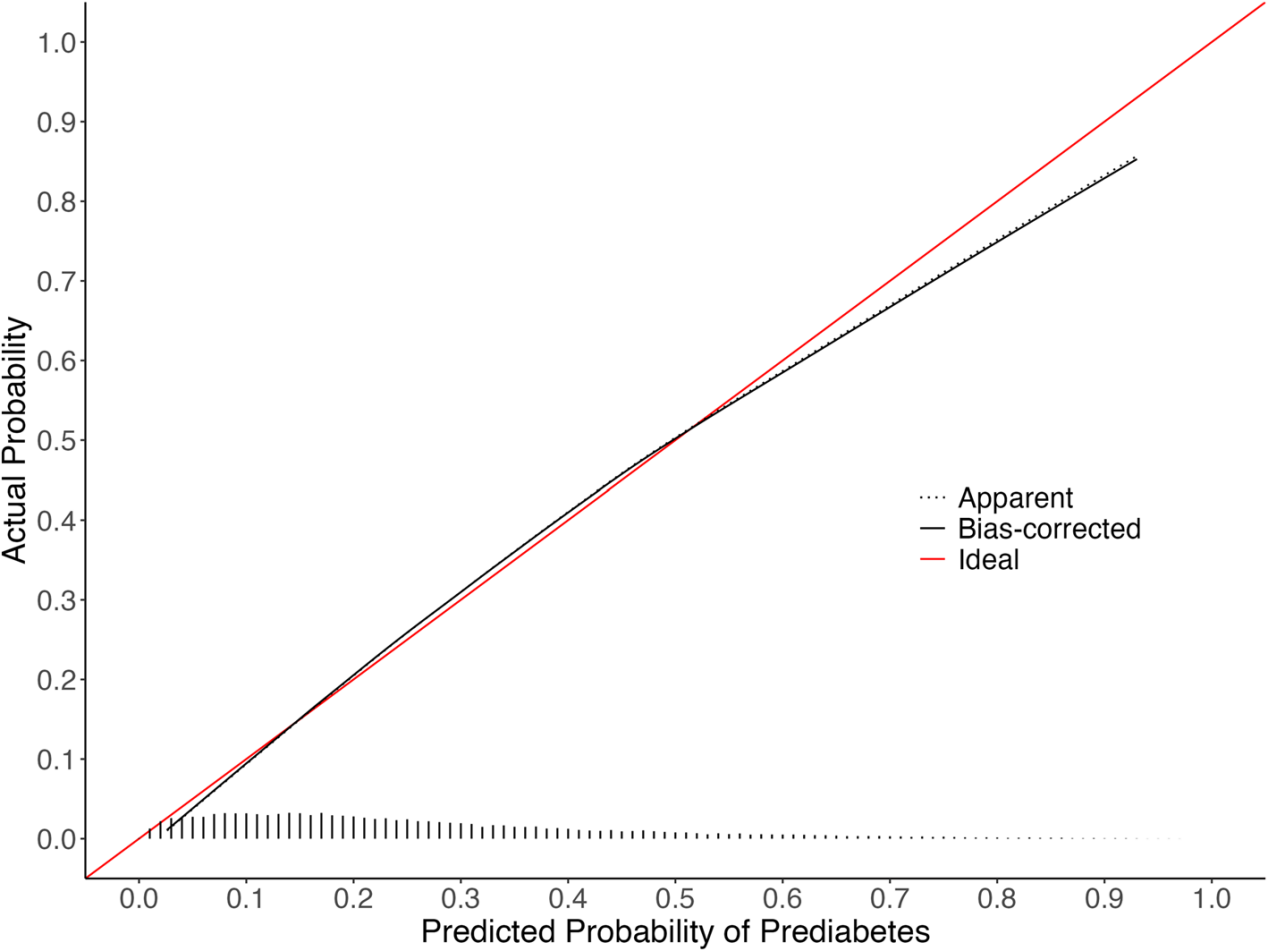
Calibration curve for approximation model fit on development EHR cohort

Descriptive statistics for the approximation model predictor variables across the development EHR and NHANES cohorts are in Table 1 (full descriptive statistics are in supplemental Tables 3–5 of Additional File 2). The LASSO model had 23 non-zero coefficients (Table 2, formula is in supplementary Table 6), with an optimism-corrected C-statistic of 0.760, intercept of -0.011, and slope of 0.987. In comparison, the approximation model had 16 coefficients (Table 2, supplemental Tables 7–10), with an optimism-corrected C-statistic of 0.763, intercept of -0.007 and a slope of 0.992. The calibration curve for LASSO revealed some underestimation for patients at moderate risk (Fig. 1). Whereas the calibration curve for the approximation model indicated overestimation for patients at high risk, the approximation was still well calibrated in the lower risk patients where the majority of the population lies (Fig. 2). The average mean absolute difference between the original LASSO model and bootstrapped predictions was 1.1%, and between the original approximation model and bootstrapped predictions was 1.8% (see additional file 2 for model instability plots).

**Table 1:** Descriptive statistics for approximation model predictor variables in EHR cohort and 2017–2020 (pre-pandemic) NHANES cohort

	EHR^a^	NHANES^a^	Weighted NHANES^1^
**N**	22,635	2,348	163,945,257
**Outcome (HbA1c ≥ 5.7%)**	5,892 (26.0%)	706 (30.1%)	36,945,826 (22.5%)
**Fasting blood glucose (mg/dL)**	96.1 (16.0)	110.5 (27.9)	109.6 (26.3)
**Smoking Status**			
Current Smoker	1,393 (23.6%)	149 (21.1%)	8,154,533 (22.1%)
Former Smoker	1,480 (25.1%)	161 (22.8%)	8,538,388 (23.1%)
Never Smoker	3,019 (51.2%)	396 (56.1%)	20,252,905 (54.8%)
**Non-HDL cholesterol (mg/dL)**	144.5 (41.7)	138.7 (41.0)	142.5 (42.4)
**Total cholesterol (mg/dL)**	191.8 (43.1)	190.5 (40.2)	195.0 (41.0)
**BMI (kg/m**^**2**^**)**	33.0 (8.4)	31.1 (7.5)	31.0 (7.5)
**eGFR (mL/min/1.73 m**^**2**^**)**	87.9 (30.8)	91.0 (20.3)	91.8 (19.6)
**Race**			
Black or African American	2,183 (37.1%)	247 (35.0%)	6,561,775 (17.8%)
Other	487 (8.3%)	243 (34.4%)	8,803,164 (23.8%)
White or Caucasian	3,222 (54.7%)	216 (30.6%)	21,580,887 (58.4%)
**Age (years)**	54.8 (14.0)	55.2 (15.7)	54.7 (15.4)

^a^Continuous variables are reported as Mean (SD) and categorical variables are reported as n (%)

**Table 2:** Model performance metrics and 95% confidence intervals for the LASSO and approximation models in the development cohort. Performance of the approximation model in 2017–2020 (pre-pandemic) NHANES is also shown

	**LASSO**^**a**^	**Approximation**^**a**^	**External validation of approximation model in NHANES**^b^	**Logistic recalibration of approximation model in NHANES**^b^	**External validation of approximation model in weighted NHANES**^b,c^	**Logistic recalibration of approximation model in weighted NHANES**^b,c^
N	22,635	22,635	2,348	2,348	2,348	2,348
Candidate variables	35	35				
Number of variables selected	23	8				
Number of coefficients	23	16	16	16	16	16
*Model Performance*						
Calibration-in-the-large (calibration intercept)	0.000 (-0.031 to 0.032)	0.000 (-0.034 to 0.034)	0.102 (0.001 to 0.199)	-0.000 (-0.097 to 0.102)	0.033 (-0.114 to 0.181)	-0.000 (-0.155 to 0.150)
Calibration slope	1.099 (1.061 to 1.141)	0.992 (0.956 to 1.029)	1.097 (0.993 to 1.209)	1.000 (0.909 to 1.099)	1.103 (0.944 to 1.274)	1.000 (0.864 to 1.151)
Brier score	0.161 (0.158 to 0.163)	0.160 (0.157 to 0.163)	0.163 (0.154 to 0.172)	0.163 (0.155 to 0.171)	0.141 (0.128 to 0.154)	0.141 (0.129 to 0.154)
ICI^d^	0.012 (0.009 to 0.016)	0.009 (0.005 to 0.013)	0.020 (0.009 to 0.036)	0.004	0.013 (0.009 to 0.037)	0.007
C-statistic	0.761 (0.755 to 0.768)	0.763 (0.756 to 0.770)	0.787 (0.768 to 0.805)	0.787 (0.767 to 0.807)	0.787 (0.765 to 0.808)	0.787 (0.766 to 0.808)

^a^Performance measures were corrected for optimism using 2,000 bootstrap resamples of the development EHR data, and the 95% confidence intervals for each optimism-corrected performance metric were derived from the 2.5th and 97.5th percentiles of these resamples

^b^95% confidence intervals were derived from the 2.5th and 97.5th percentiles of 2,000 bootstrap resamples of the NHANES data

^c^The performance metrics for the weighted NHANES were weighted using normalized fasting subsample weights

^d^The integrated calibration index (ICI) is the average absolute difference between the predicted probabilities and observed probabilities derived from the locally weighted scatter plot smoother (LOWESS). For the weighted NHANES, the loess function was used with the normalized fasting subsample weights specified for the weights argument

**Fig. 3:**
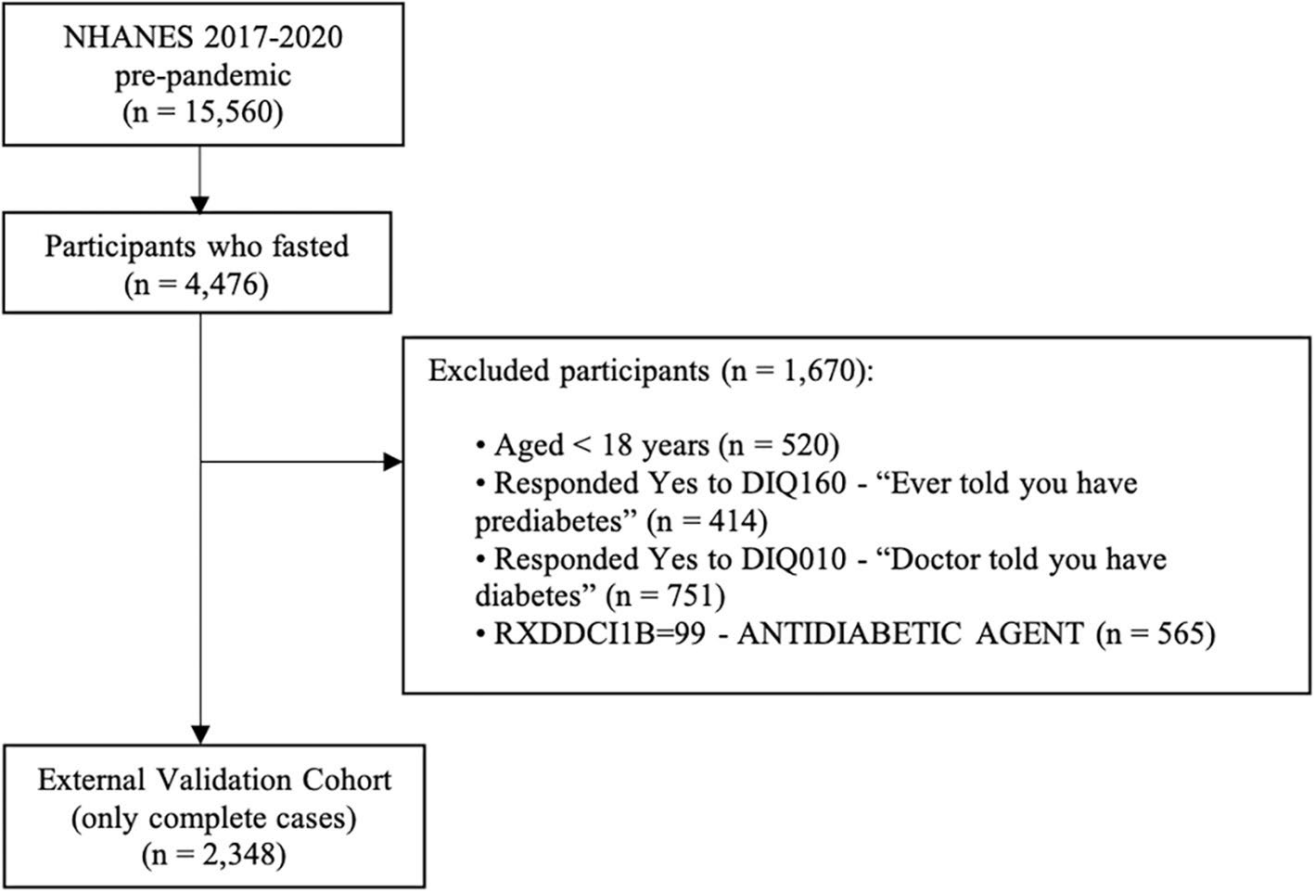
Flow diagram for 2017–2020 (pre-pandemic) NHANES external validation cohort

**Fig. 4:**
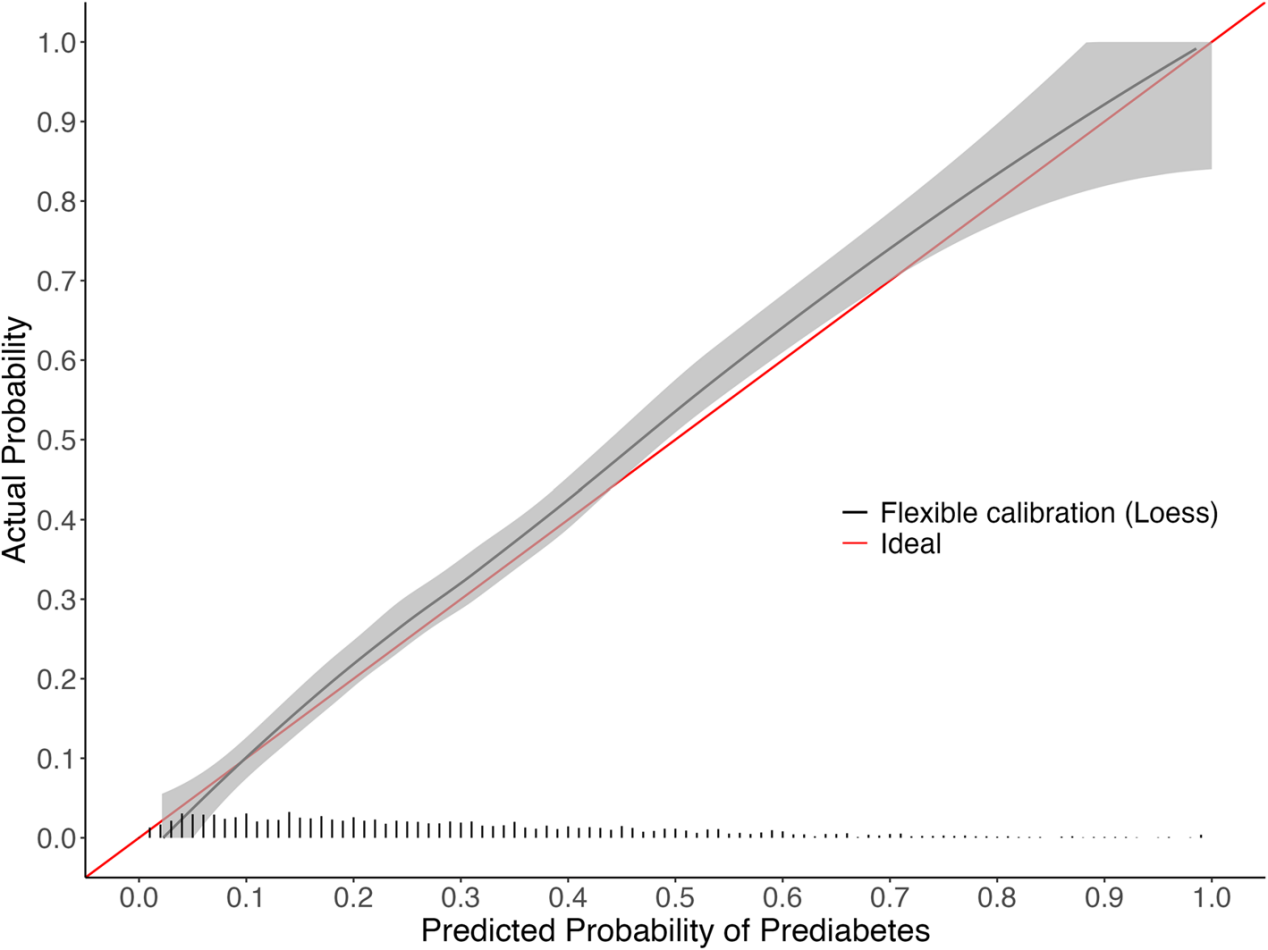
Calibration curve after applying the approximation model coefficients to NHANES

**Fig. 5:**
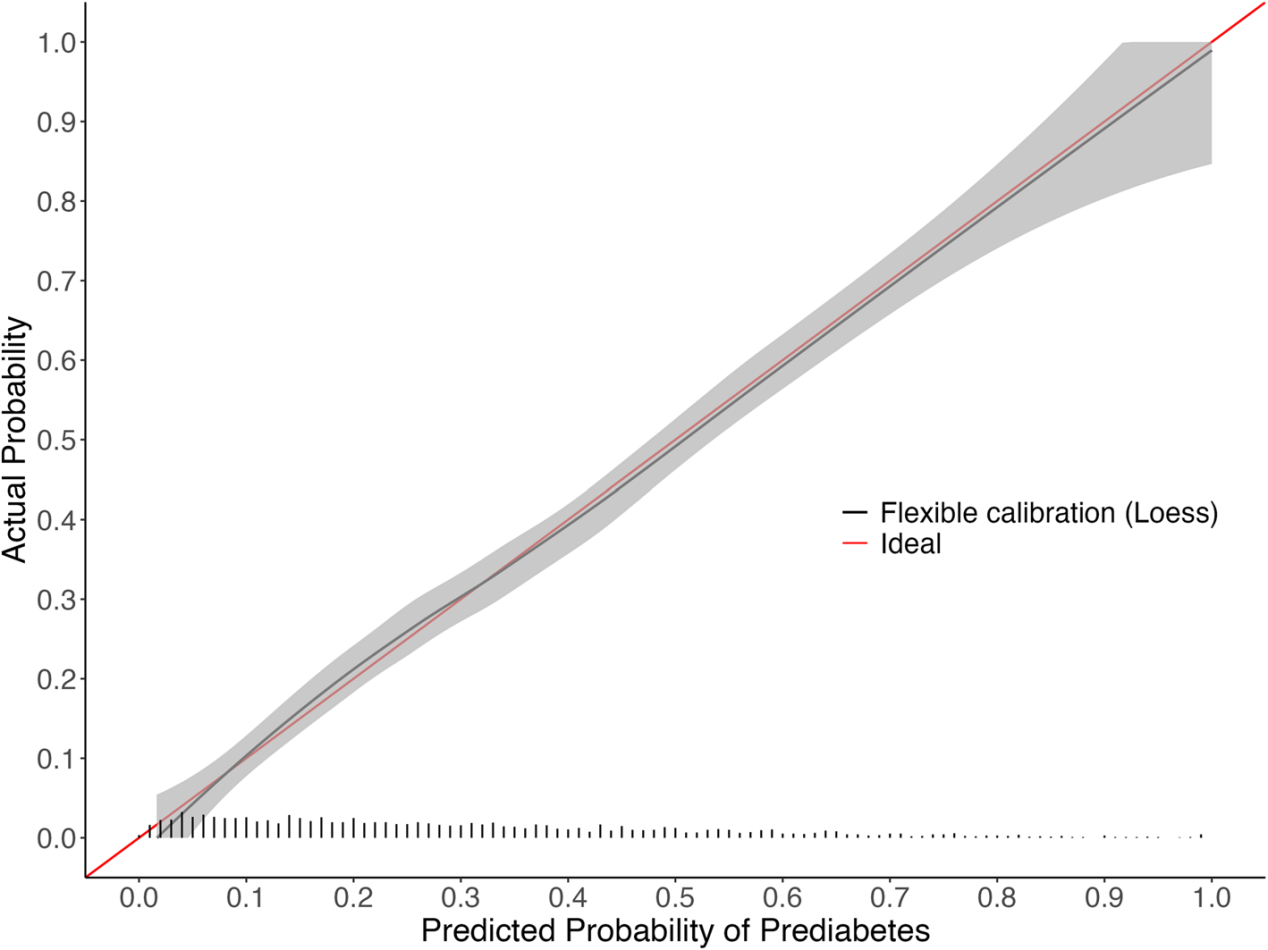
Calibration curve after recalibrating the approximation model to NHANES

**Fig. 6:**
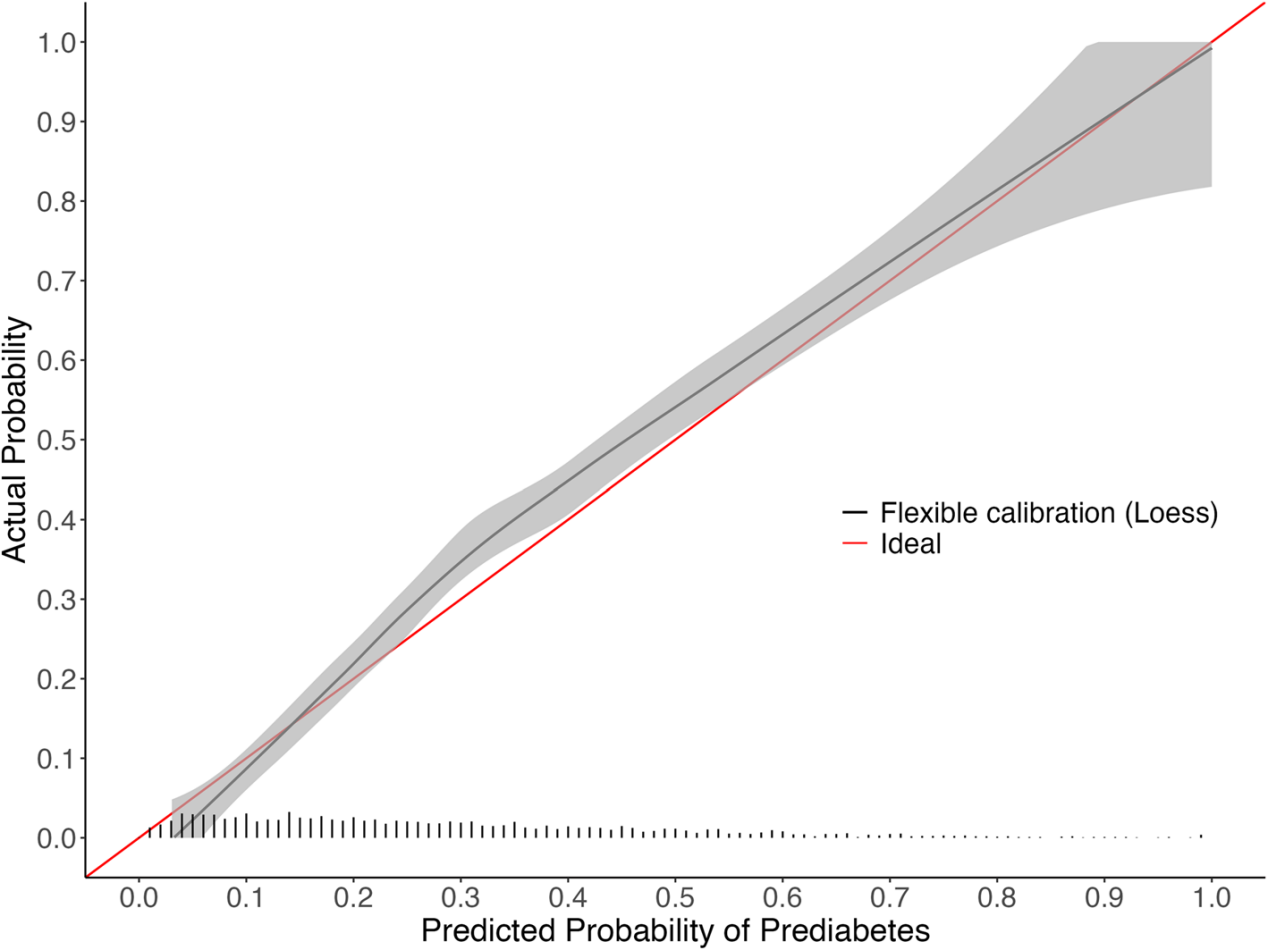
Calibration curve after applying the approximation model coefficients to NHANES with normalized fasting subsample weights

**Fig. 7:**
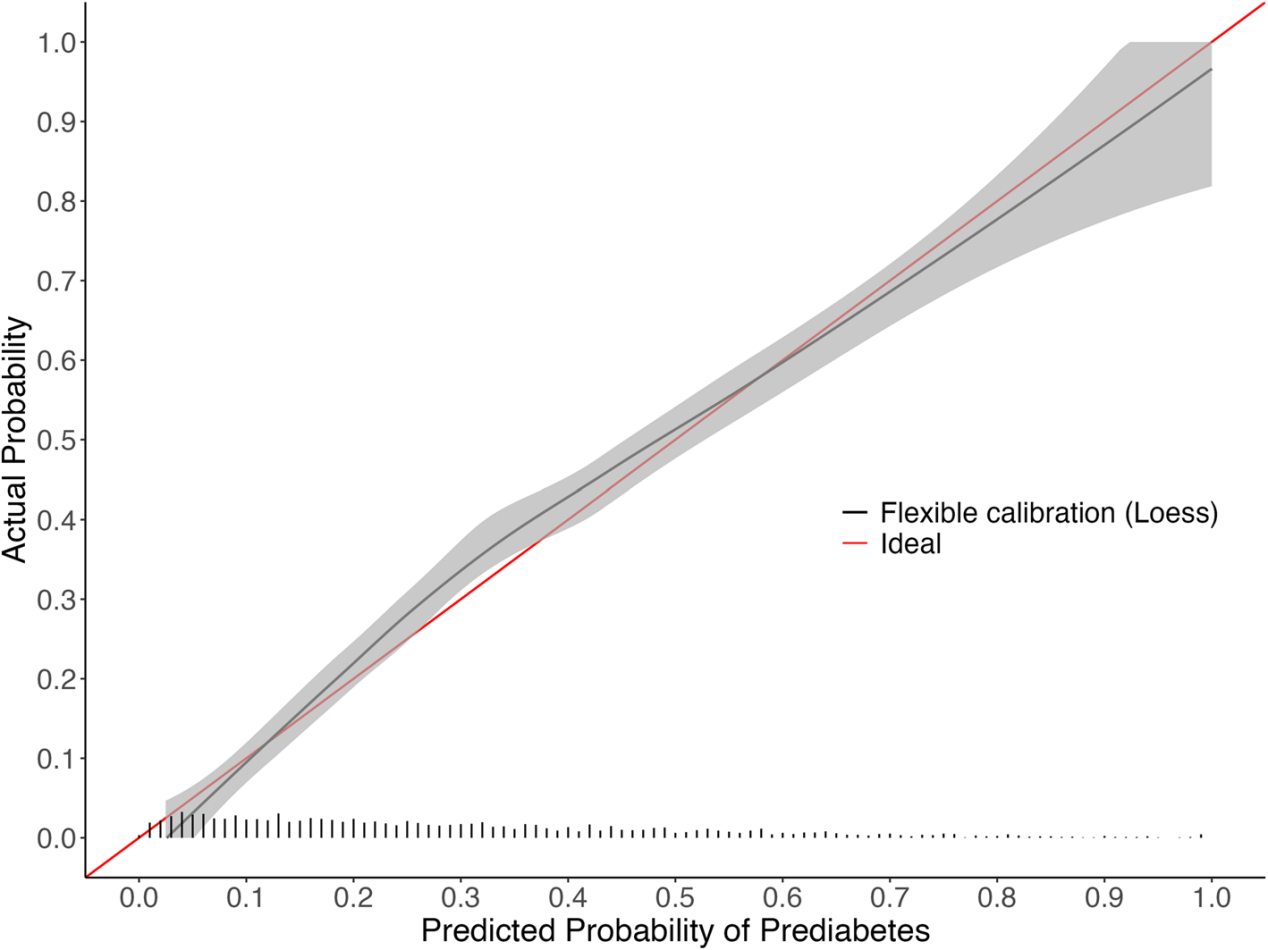
Calibration curve after recalibrating the approximation model to NHANES with normalized fasting subsample weights

The NHANES validation cohort included 2,348 participants (Fig. 3). A larger proportion of participants (30.1%) had an elevated HbA1c in NHANES compared to 26.0% of patients in the development cohort. The means for continuous approximation predictors among participants with prediabetes between the development and NHANES cohort were similar (age 54.8 years vs 55.5 years; BMI 33.0% vs 31.1%; non-HDL 144 mg/dL vs 138.7 mg/dL; total cholesterol 192 mg/dL vs 191 mg/dL). The proportion of Black or African American participants was slightly higher (37.1% vs 35%), and the proportion of current smokers was similar (23.6% vs 21.1%), respectively. The approximation model had a C-statistic of 0.787 when applied to NHANES and showed miscalibration and overestimation (Table 2 and Fig. 4; Intercept 0.102; Slope 1.097; ICI 0.020), but the calibration improved after recalibration (Table 2 and Fig. 5; Intercept -0.000; Slope 1; ICI 0.004). Calibration of the approximation model in NHANES modestly improved with use of normalized fasting subsample weights (Table 2 and Fig. 6; Intercept 0.033; Slope 1.103; ICI 0.013). The formulas for the approximation model recalibrated in NHANES with and without sampling weights are available in supplemental Tables 9 and 10. (Fig. 7).

## Discussion

Both the LASSO and approximation models demonstrated moderate discriminative performance in the development cohort, with the approximation model showing better calibration than the LASSO in development, and improved discrimination in the external validation cohort. The approximation model performed similarly to the LASSO model in the development cohort despite differences in variable selection methods. The LASSO uses shrinkage for variable selection by constraining the absolute values of the regression coefficients to be less than a bound determined by a penalty parameter lambda, effectively zeroing out coefficients of less relevant predictors to simplify the model [16, 32]. In contrast, the stepdown/backwards elimination method approximates the full logistic regression model by retaining the most significant predictors that explain the majority of the variance. This process results in a parsimonious model that effectively captures the key predictive relationships, maintaining similar performance to the full model. The approximation method can lead to a model that is easier to interpret which is beneficial in settings where simplicity and clarity are prioritized. The LASSO method can lead to a more simplified model that reduces overfitting, improves generalizability, and may enhance predictive performance, especially when dealing with large datasets with many predictors.

When applied to NHANES data, the approximation model showed good discriminative ability in predicting elevated HbA1c levels. Recalibration further improved its performance, indicating that some tuning to specific populations can enhance the model's utility for identifying individuals with an HbA1c ≥ 5.7%. The logistic recalibration allowed us to maintain the knowledge collected from the development data while incorporating knowledge from new patient data. We did not see the need for refitting the approximation model in NHANES, as refitting potentially disregards the knowledge gained from the development data [34, 35]. The use of normalized fasting subsample weights had negligible impact on the approximation model’s performance in NHANES, as normalization scales the weights so that their sum equals the sample size, which does not significantly influence individual predictions.

Using surveys or epidemiological cohort studies for EHR model validation, and vice versa, can be challenging due to temporal changes in disease prevalence and differences in variable measurement [36]. For instance, the CHARGE-AF risk score for atrial fibrillation was developed and validated using multiple prospective cohort studies, yet performed poorly when it was validated with single-institution EHR [36, 37]. Cohort studies may also have strict inclusion and exclusion criteria, and models developed using such data may have limited generalizability [27]. Moreover, models developed from routinely-collected EHR data may not perform well on cohort data because of differences in data collection, in patient catchment, and in overall outcome and predictor variable occurrence [38–40]. Accordingly, it is recommended that external validation be performed in new data that is relevant to the model’s intended use [14, 15]. While the approximation model is designed to predict elevated HbA1c in a clinical setting, NHANES participants, though originating from a different context than the development data, are relevant because they represent the underlying target superpopulation of patients with prediabetes.

Healthcare organizations with limited resources or smaller populations may benefit from applying the original coefficients described by Wells et al., as this can be implemented using a calculator outside the EHR, while those needing a predefined parsimonious model but lacking the capacity to capture all original variables may prefer recalibrating the approximation model. If data extraction and refitting are feasible, updating the approximation model's coefficients could enhance its discriminative ability. Complete full model refitting and approximation might be desirable for organizations wanting to deploy a prediction model that is more rigorously fit to local patient characteristics and available data, especially if the population is considerably different from the AHWFBMC EHR and NHANES cohorts as demonstrated in Alhassan et al. [11].

### Strengths and Limitations

Strengths of our study include the use of a relatively large sample size for model development and the application of bootstrap resampling for internal validation and evaluation of predictive performance. The external validation was limited by a smaller sample size of the NHANES validation cohort, differences in variable definitions between the development cohort and NHANES, and the inability to validate the LASSO model. Although NHANES includes many of the structured data elements found in EHRs, it lacked some of the disease states associated with diabetes risk. To ensure consistent classification of variables, we used International Classification of Diseases 10th Revision (ICD-10) codes in the NHANES drug files to capture diagnoses whenever possible and minimized the use of questionnaire variables.

## Conclusion

We showed that an approximation model intended to identify patients with an elevated HbA1c exhibited adequate predictive performance among an external population. The results indicate that the model may be transportable across different settings, making it a valuable and resource-efficient tool in clinical practice for identifying patients with prediabetes who could benefit from early intervention to prevent disease progression and adverse outcomes. Further validating the approximation model with external EHR data would strengthen evidence of the model’s transportability and generalizability, and provide insights into optimizing its integration within different EHR systems for use in routine clinical practice.

## Supplementary Information

Supplementary Material 1.

Supplementary Material 2.

Supplementary Material 3.

## Data Availability

The data files analyzed in this study are publicly available on the NHANES website at https://wwwn.cdc.gov/nchs/nhanes/Default.aspx. The development dataset and code used for this study is available from the corresponding author upon reasonable request. A study protocol was not prepared.

## Abbreviations

ADA: American Diabetes Association
BMI: Body mass index
CDC: Centers for Disease Control and Prevention
CKD-EPI: Chronic Kidney Disease Epidemiology Collaboration
C-statistic: Concordance statistic
eGFR: Estimated glomerular filtration rate
EHR: Electronic health record
HbA1c: Glycated hemoglobin
HDL: High-density lipoprotein
NHANES: National Health and Nutrition Examination Survey
non-HDL: Non–high-density lipoprotein
SBP: Systolic blood pressure
SCr: Serum creatinine
ICI: Integrated calibration index
