# Supplementary material for: Development, validation and recalibration of a prediction model for prediabetes: an EHR and NHANES-based study
Source: BMC Med Inform Decis Mak. 2024 Dec 18;24:387. doi: 10.1186/s12911-024-02803-w (PMC11657225; doi:10.1186/s12911-024-02803-w)
Supplement: Supplementary file 1 — Supplementary Material 1. [file 12911_2024_2803_MOESM1_ESM.docx]

**Additional file 1.** Data Dictionary

| **Development Cohort** | **Validation Cohort (NHANES 2017-2020)** |
| --- | --- |
| **Laboratory measurements** | |
| Serum triglycerides | LBXTR |
| Random blood glucose | LBXGLU |
| Serum non–high-density lipoprotein | LBXTC - LBDHDD |
| Serum high-density lipoprotein (dropped due to an inability to fit model) | LBDHDD |
| Serum total cholesterol | LBXTC |
| Estimated glomerular filtration rate | estimated from LBXSCR using the Chronic Kidney Disease Epidemiology Collaboration (CKD-EPI) Creatinine Equation (2021) without race |
| **Active prescription medication categories** | |
| Antihypertensive | RXDRSC1 ="I10.P" or "I10", or RXDRSC2 = "I10.P" or "I10", BUT NOT RXDDRGID = "d00144" |
| First generation antipsychotic | RXDDRGID = "d00064", "d00027", or "d00855" |
| Second generation antipsychotic | RXDDCI1C = 341 |
| 3-hydroxy-3-methyl-glutaryl-coenzyme A reductase inhibitor (statin) | RXDDCI1C = 173 or 317 |
| Fibrate | RXDDCI1C = 241 |
| Valproic acid | RXDDRGID = "d00083" or "d03833" |
| Beta-blocker | RXDDCI1B = 47 |
| Thiazide diuretic | RXDDCI1C = 156 or 467 |
| Niacin | RXDDRGID = "d00314", "d07110", or "d04787" |
| Oral glucocorticoid | RXDDCI1B = 98 |
| Protease inhibitor | RXDDCI1C = 175 or RXDDRGID = "d08305" or "d04717" |
| Nucleoside reverse transcriptase inhibitor | RXDDCI1C = 176 or RXDDRGID = "d08284", "d05354", "d04727", "d07899", "d05847", "d07796", "d05352", or "d04219" |
| Oral contraceptive | RXDDCI1C = 102 BUT NOT RXDDRGID = "d04772", "d04773", or "d00557" |
| Injectable medroxyprogesterone acetate | RXDDRGID = "d03819", "d04721", or "d00284" |
| Cyclosporine | RXDDRGID = "d00079" |
| Tacrolimus | RXDDRGID = "d03752" |
| **Diagnosis codes** | |
| Hypertension | BPQ050A = 1 or taking antihypertensive |
| Ischemic heart disease | RXDRSC1or RXDRSC2 in "I20” through “I25” |
| Peripheral vascular disease | NOT AVAILABLE |
| Neuropathy | NOT AVAILABLE |
| Obesity | BMXBMI >= 30.0 |
| Hyperlipidemia | RXDRSC1 = "E78.0" or "E78.0P", or RXDRSC2 = "E78.1", or RXDDCI1B=19, or BPQ100D=1 |
| **Vital signs** | |
| Systolic blood pressure | mean of BPXSY1, BPXSY2, BPXSY3 |
| Diastolic blood pressure | mean of BPXDI1, BPXDI2, BPXDI3 |
| Body mass index | BMXBMI |
| **Demographics** | |
| Race | White or Caucasian: RIDRETH3 = 3 Black or African American: RIDRETH3 = 4 Other: RIDRETH3 = 1, 2, 6, and 7 |
| Age | RIDAGEYR |
| Gender | RIAGENDR |
| **Family history** | |
| Number of first degree relatives with diabetes | (Close relative had diabetes?)  MCQ300c = 1 |
| **Social history** | |
| Smoking status | Current Smoker: SMQ020 = 1 AND SMQ040 = 1 or 2 Former Smoker: SMQ020 = 1 AND SMQ040 = 3 Never Smoker: SMQ020 = 2 |
