# Supplementary material for: Development, validation and recalibration of a prediction model for prediabetes: an EHR and NHANES-based study
Source: BMC Med Inform Decis Mak. 2024 Dec 18;24:387. doi: 10.1186/s12911-024-02803-w (PMC11657225; doi:10.1186/s12911-024-02803-w)

| **Supplemental Table 3.** Descriptive statistics for Development EHR cohort, by prediabetes status. | | | |
| --- | --- | --- | --- |
| **Characteristics** | **Total**  N = 22635*^1^* | **HbA1c <5.7%**  N = 16743 (74.0%)*^1^* | **HbA1c ≥5.7%**  N = 5892 (26.0%)*^1^* |
| **Age (years)** | 49.8 (15.4) | 48.1 (15.4) | 54.8 (14.0) |
| **Gender** |  |  |  |
| Female | 14,202 (62.7%) | 10,666 (63.7%) | 3,536 (60.0%) |
| Male | 8,433 (37.3%) | 6,077 (36.3%) | 2,356 (40.0%) |
| **Race** |  |  |  |
| Black or African American | 5,875 (26.0%) | 3,692 (22.1%) | 2,183 (37.1%) |
| Other | 1,665 (7.4%) | 1,178 (7.0%) | 487 (8.3%) |
| White or Caucasian | 15,095 (66.7%) | 11,873 (70.9%) | 3,222 (54.7%) |
| **Smoking status** |  |  |  |
| Current Smoker | 4,140 (18.3%) | 2,747 (16.4%) | 1,393 (23.6%) |
| Former Smoker | 5,347 (23.6%) | 3,867 (23.1%) | 1,480 (25.1%) |
| Never Smoker | 13,148 (58.1%) | 10,129 (60.5%) | 3,019 (51.2%) |
| **Fasting blood glucose (mg/dL)** | 90.4 (14.1) | 88.4 (12.7) | 96.1 (16.0) |
| **Triglycerides (mg/dL)** | 133.4 (96.7) | 127.4 (89.0) | 150.4 (114.2) |
| **Non-HDL cholesterol (mg/dL)** | 137.5 (38.8) | 135.0 (37.4) | 144.5 (41.7) |
| **Total cholesterol (mg/dL)** | 187.5 (40.5) | 186.0 (39.4) | 191.8 (43.1) |
| **BMI (kg/m²)** | 30.9 (7.8) | 30.1 (7.4) | 33.0 (8.4) |
| **Obesity** | 4,002 (17.7%) | 2,997 (17.9%) | 1,005 (17.1%) |
| **eGFR (mL/min/1.73 m²)** | 90.9 (32.5) | 91.9 (33.0) | 87.9 (30.8) |
| **Systolic Blood Pressure (mmHg)** | 131.2 (18.2) | 129.6 (17.7) | 135.5 (19.1) |
| **Diastolic Blood Pressure (mmHg)** | 76.6 (11.6) | 76.0 (11.4) | 78.2 (11.9) |
| **Hypertension** | 8,050 (35.6%) | 5,569 (33.3%) | 2,481 (42.1%) |
| **Hyperlipidemia** | 5,452 (24.1%) | 3,901 (23.3%) | 1,551 (26.3%) |
| **Ischemic heart disease** | 1,486 (6.6%) | 1,016 (6.1%) | 470 (8.0%) |
| **Peripheral Vascular Disease** | 215 (0.9%) | 139 (0.8%) | 76 (1.3%) |
| **Neuropathy** | 283 (1.3%) | 216 (1.3%) | 67 (1.1%) |
| **Number of first degree relatives with diabetes** |  |  |  |
| 0 | 16,371 (72.3%) | 12,319 (73.6%) | 4,052 (68.8%) |
| 1 | 5,376 (23.8%) | 3,848 (23.0%) | 1,528 (25.9%) |
| 2 or 3 | 888 (3.9%) | 576 (3.4%) | 312 (5.3%) |
| **Statin** | 2,645 (11.7%) | 1,738 (10.4%) | 907 (15.4%) |
| **Beta-blocker** | 2,358 (10.4%) | 1,606 (9.6%) | 752 (12.8%) |
| **Thiazide diuretic** | 2,502 (11.1%) | 1,527 (9.1%) | 975 (16.5%) |
| **Oral glucocorticoid** | 388 (1.7%) | 304 (1.8%) | 84 (1.4%) |
| **Antihypertensive** | 6,535 (28.9%) | 4,417 (26.4%) | 2,118 (35.9%) |
| **Fibrates** | 199 (0.9%) | 141 (0.8%) | 58 (1.0%) |
| **Niacin** | 123 (0.5%) | 82 (0.5%) | 41 (0.7%) |
| **Valproic Acid** | 175 (0.8%) | 134 (0.8%) | 41 (0.7%) |
| **First generation antipsychotic** | 22 (0.1%) | 16 (0.1%) | 6 (0.1%) |
| **Second generation antipsychotic** | 696 (3.1%) | 538 (3.2%) | 158 (2.7%) |
| **Protease inhibitor** | 51 (0.2%) | 35 (0.2%) | 16 (0.3%) |
| **Nucleoside reverse transcriptase inhibitor** | 89 (0.4%) | 62 (0.4%) | 27 (0.5%) |
| **Oral contraceptive** | 923 (4.1%) | 846 (5.1%) | 77 (1.3%) |
| **Injectable medroxyprogesterone acetate** | 90 (0.4%) | 74 (0.4%) | 16 (0.3%) |
| **Cyclosporine** | 21 (0.1%) | 18 (0.1%) | 3 (0.1%) |
| **Tacrolimus** | 139 (0.6%) | 128 (0.8%) | 11 (0.2%) |
| *^1^*Continuous variables are reported as Mean (SD) and categorical variables are reported as n (%). | | | |

| **Supplemental Table 4.** Descriptive statistics for NHANES 2017-2020 pre-pandemic sample, by prediabetes status. | | | |
| --- | --- | --- | --- |
| **Characteristics** | **Total**  N = 2348*^1^* | **HbA1c <5.7%**  N = 1642 (69.9%)*^1^* | **HbA1c ≥5.7%**  N = 706 (30.1%)*^1^* |
| **Age (years)** | 47.5 (17.3) | 44.1 (16.9) | 55.2 (15.7) |
| **Gender** |  |  |  |
| Female | 1,205 (51.3%) | 872 (53.1%) | 333 (47.2%) |
| Male | 1,143 (48.7%) | 770 (46.9%) | 373 (52.8%) |
| **Race** |  |  |  |
| Black or African American | 564 (24.0%) | 317 (19.3%) | 247 (35.0%) |
| Other | 912 (38.8%) | 669 (40.7%) | 243 (34.4%) |
| White or Caucasian | 872 (37.1%) | 656 (40.0%) | 216 (30.6%) |
| **Smoking status** |  |  |  |
| Current Smoker | 452 (19.3%) | 303 (18.5%) | 149 (21.1%) |
| Former Smoker | 503 (21.4%) | 342 (20.8%) | 161 (22.8%) |
| Never Smoker | 1,393 (59.3%) | 997 (60.7%) | 396 (56.1%) |
| **Fasting blood glucose (mg/dL)** | 102.4 (18.0) | 98.8 (9.4) | 110.5 (27.9) |
| **Triglycerides (mg/dL)** | 102.6 (89.1) | 98.5 (94.4) | 112.3 (74.7) |
| **Non-HDL cholesterol (mg/dL)** | 131.6 (39.8) | 128.5 (38.9) | 138.7 (41.0) |
| **Total cholesterol (mg/dL)** | 186.5 (40.2) | 184.8 (40.1) | 190.5 (40.2) |
| **BMI (kg/m²)** | 29.0 (7.0) | 28.1 (6.6) | 31.1 (7.5) |
| **Obesity** |  |  |  |
| FALSE | 1,488 (63.4%) | 1,123 (68.4%) | 365 (51.7%) |
| TRUE | 860 (36.6%) | 519 (31.6%) | 341 (48.3%) |
| **eGFR (mL/min/1.73 m²)** | 97.1 (20.7) | 99.8 (20.3) | 91.0 (20.3) |
| **Systolic Blood Pressure (mmHg)** | 121.9 (18.0) | 119.4 (16.9) | 127.7 (19.3) |
| **Diastolic Blood Pressure (mmHg)** | 74.3 (11.3) | 73.3 (11.0) | 76.6 (11.8) |
| **Hypertension** | 519 (22.1%) | 277 (16.9%) | 242 (34.3%) |
| **Hyperlipidemia** | 368 (15.7%) | 183 (11.1%) | 185 (26.2%) |
| **Ischemic heart disease** | 122 (5.2%) | 66 (4.0%) | 56 (7.9%) |
| **Close relative had diabetes?** |  |  |  |
| Don't know | 31 (1.3%) | 25 (1.5%) | 6 (0.8%) |
| No | 1,359 (57.9%) | 985 (60.0%) | 374 (53.0%) |
| Yes | 958 (40.8%) | 632 (38.5%) | 326 (46.2%) |
| **Statin** | 305 (13.0%) | 148 (9.0%) | 157 (22.2%) |
| **Beta-blocker** | 214 (9.1%) | 111 (6.8%) | 103 (14.6%) |
| **Thiazide diuretic** | 95 (4.0%) | 53 (3.2%) | 42 (5.9%) |
| **Oral glucocorticoid** | 36 (1.5%) | 24 (1.5%) | 12 (1.7%) |
| **Antihypertensive** | 461 (19.6%) | 241 (14.7%) | 220 (31.2%) |
| **Fibrates** | 17 (0.7%) | 8 (0.5%) | 9 (1.3%) |
| **Niacin** | 1 (0.0%) | 1 (0.1%) | 0 (0.0%) |
| **Valproic Acid** | 9 (0.4%) | 6 (0.4%) | 3 (0.4%) |
| **First generation antipsychotic** | 1 (0.0%) | 0 (0.0%) | 1 (0.1%) |
| **Second generation antipsychotic** | 35 (1.5%) | 26 (1.6%) | 9 (1.3%) |
| **Protease inhibitor** | 4 (0.2%) | 2 (0.1%) | 2 (0.3%) |
| **Nucleoside reverse transcriptase inhibitor** | 7 (0.3%) | 5 (0.3%) | 2 (0.3%) |
| **Oral contraceptive** | 45 (1.9%) | 41 (2.5%) | 4 (0.6%) |
| **Injectable medroxyprogesterone acetate** | 3 (0.1%) | 3 (0.2%) | 0 (0.0%) |
| **Cyclosporine** | 0 (0.0%) | 0 (0.0%) | 0 (0.0%) |
| **Tacrolimus** | 2 (0.1%) | 1 (0.1%) | 1 (0.1%) |
| *^1^*Continuous variables are reported as Mean (SD) and categorical variables are reported as n (%). | | | |

| **Supplemental Table 5.** Descriptive statistics for NHANES 2017-2020 pre-pandemic weighted sample, by prediabetes status. | | | |
| --- | --- | --- | --- |
| **Characteristics** | **Total**  N = 163945257 *^1^* | **HbA1c <5.7%**  N = 126999431 (77.5%)*^1^* | **HbA1c ≥5.7%**  N = 36945826 (22.5%)*^1^* |
| **Age (years)** | 45.7 (17.0) | 43.0 (16.5) | 54.7 (15.4) |
| **Gender** |  |  |  |
| Female | 83,184,874 (50.7%) | 63,285,942 (49.8%) | 19,898,932 (53.9%) |
| Male | 80,760,383 (49.3%) | 63,713,489 (50.2%) | 17,046,894 (46.1%) |
| **Race** |  |  |  |
| Black or African American | 17,546,044 (10.7%) | 10,984,270 (8.6%) | 6,561,775 (17.8%) |
| Other | 40,350,078 (24.6%) | 31,546,913 (24.8%) | 8,803,164 (23.8%) |
| White or Caucasian | 106,049,135 (64.7%) | 84,468,248 (66.5%) | 21,580,887 (58.4%) |
| **Smoking status** |  |  |  |
| Current Smoker | 27,490,180 (16.8%) | 19,335,648 (15.2%) | 8,154,533 (22.1%) |
| Former Smoker | 40,465,265 (24.7%) | 31,926,877 (25.1%) | 8,538,388 (23.1%) |
| Never Smoker | 95,989,811 (58.5%) | 75,736,906 (59.6%) | 20,252,905 (54.8%) |
| **Fasting blood glucose (mg/dL)** | 101.7 (15.5) | 99.4 (9.3) | 109.6 (26.3) |
| **Triglycerides (mg/dL)** | 102.8 (88.2) | 97.9 (88.9) | 119.6 (83.4) |
| **Non-HDL cholesterol (mg/dL)** | 131.8 (40.3) | 128.7 (39.2) | 142.5 (42.4) |
| **Total cholesterol (mg/dL)** | 187.6 (40.3) | 185.4 (39.8) | 195.0 (41.0) |
| **BMI (kg/m²)** | 28.7 (6.9) | 28.0 (6.5) | 31.0 (7.5) |
| **Obesity** |  |  |  |
| FALSE | 107,970,659 (65.9%) | 88,373,936 (69.6%) | 19,596,723 (53.0%) |
| TRUE | 55,974,598 (34.1%) | 38,625,495 (30.4%) | 17,349,103 (47.0%) |
| **eGFR (mL/min/1.73 m²)** | 98.7 (19.9) | 100.7 (19.5) | 91.8 (19.6) |
| **Systolic Blood Pressure (mmHg)** | 119.7 (16.2) | 118.1 (15.4) | 125.0 (17.9) |
| **Diastolic Blood Pressure (mmHg)** | 73.4 (10.7) | 72.8 (10.5) | 75.4 (11.3) |
| **Hypertension** | 30,930,382 (18.9%) | 18,577,000 (14.6%) | 12,353,382 (33.4%) |
| **Hyperlipidemia** | 24,794,898 (15.1%) | 14,539,203 (11.4%) | 10,255,695 (27.8%) |
| **Ischemic heart disease** | 6,530,577 (4.0%) | 3,839,600 (3.0%) | 2,690,977 (7.3%) |
| **Close relative had diabetes?** |  |  |  |
| Don't know | 2,312,722 (1.4%) | 2,151,267 (1.7%) | 161,455 (0.4%) |
| No | 96,564,712 (58.9%) | 77,676,634 (61.2%) | 18,888,078 (51.1%) |
| Yes | 65,067,823 (39.7%) | 47,171,530 (37.1%) | 17,896,293 (48.4%) |
| **Statin** | 20,311,054 (12.4%) | 11,914,200 (9.4%) | 8,396,854 (22.7%) |
| **Beta-blocker** | 13,834,316 (8.4%) | 8,452,050 (6.7%) | 5,382,266 (14.6%) |
| **Thiazide diuretic** | 6,770,778 (4.1%) | 4,526,555 (3.6%) | 2,244,223 (6.1%) |
| **Oral glucocorticoid** | 2,166,209 (1.3%) | 1,629,550 (1.3%) | 536,660 (1.5%) |
| **Antihypertensive** | 27,308,924 (16.7%) | 16,167,520 (12.7%) | 11,141,404 (30.2%) |
| **Fibrates** | 1,546,771 (0.9%) | 507,993 (0.4%) | 1,038,778 (2.8%) |
| **Niacin** | 248,585 (0.2%) | 248,585 (0.2%) | 0 (0.0%) |
| **Valproic** | 716,668 (0.4%) | 624,046 (0.5%) | 92,623 (0.3%) |
| **First generation antipsychotic** | 18,680 (0.0%) | 0 (0.0%) | 18,680 (0.1%) |
| **Second generation antipsychotic** | 2,493,356 (1.5%) | 1,822,301 (1.4%) | 671,056 (1.8%) |
| **Protease inhibitor** | 120,859 (0.1%) | 68,995 (NA%) | 51,864 (NA%) |
| **Nucleoside reverse transcriptase inhibitor** | 227,992 (0.1%) | 176,128 (0.1%) | 51,864 (0.1%) |
| **Oral contraceptive** | 4,139,367 (2.5%) | 3,791,272 (3.0%) | 348,095 (0.9%) |
| **Injectable medroxyprogesterone acetate** | 144,507 (0.1%) | 144,507 (0.1%) | 0 (0.0%) |
| **Cyclosporine** | 0 (0.0%) | 0 (0.0%) | 0 (0.0%) |
| **Tacrolimus** | 19,423 (0.0%) | 12,952 (0.0%) | 6,471 (0.0%) |
| *^1^*Continuous variables are reported as Mean (SD) and categorical variables are reported as n (%). | | | |

| **Supplemental Table 6**. LASSO logistic regression for predicting the probability of glycated hemoglobin ≥5.7%. | |
| --- | --- |
| N = 22635 | **Coefficient (S.E.)** |
| Intercept | -7.9395 (0.2459) |
| Triglycerides (mg/dL) | 0.0012 (0.0002) |
| Fasting blood glucose (mg/dL) | 0.0316 (0.0012) |
| Antihypertensive | 0.0000 (0.0587) |
| Second generation antipsychotic | -0.0042 (0.0986) |
| Smoking Status=Former Smoker | -0.2549 (0.0509) |
| Smoking Status=Never Smoker | -0.3419 (0.0447) |
| Non-HDL cholesterol (mg/dL) | 0.0031 (0.0014) |
| First generation antipsychotic | 0.0000 (0.5348) |
| Statin | 0.1310 (0.0570) |
| Fibrates | 0.0000 (0.1680) |
| Valproic acid | 0.0000 (0.1917) |
| Beta-blocker | 0.0000 (0.0620) |
| Thiazide diuretic | 0.2003 (0.0600) |
| Niacin | 0.0000 (0.2090) |
| Oral glucocorticoid | 0.0000 (0.1380) |
| Protease inhibitor | 0.0000 (0.3437) |
| Nucleoside reverse transcriptase inhibitor | 0.0000 (0.2541) |
| Oral contraceptive | -0.3014 (0.1173) |
| Injectable medroxyprogesterone acetate | 0.0000 (0.2861) |
| Cyclosporine | 0.0000 (0.6265) |
| Tacrolimus | -0.4488 (0.2837) |
| eGFR (mL/min/1.73 m²) | 0.0024 (0.0008) |
| Hypertension | -0.1340 (0.0479) |
| Ischemic heart disease | 0.0000 (0.0692) |
| Peripheral Vascular Disease | 0.0000 (0.1589) |
| Neuropathy | -0.1917 (0.1442) |
| Obesity | -0.2769 (0.0487) |
| Hyperlipidemia | -0.0415 (0.0456) |
| Race=Other | 0.0000 (0.0680) |
| Race=White or Caucasian | -0.8928 (0.0394) |
| Age (years) | 0.0364 (0.0016) |
| Number of first degree relatives with diabetes=1 | 0.0952 (0.0387) |
| Number of first degree relatives with diabetes=2 or 3 | 0.0816 (0.0791) |
| BMI (kg/m²) | 0.0546 (0.0025) |
| Gender=Male | 0.1061 (0.0482) |
| Total cholesterol (mg/dL) | 0.0000 (0.0013) |
| Systolic Blood Pressure (mmHg) | 0.0007 (0.0011) |
| Diastolic Blood Pressure (mmHg) | 0.0042 (0.0018) |
| The linear predictor for the LASSO model from the development cohort is as follows: -7.939522371 + 0.001160328 * tg + 0.031576342 * redo.glu - 0 * current.antihtn - 0.004171066 * antipsych.2 - 0.254856432 * (smoking.status=="Former Smoker") - 0.34186959 *(smoking.status=="Never Smoker") + 0.003050742 * non.hdl - 0 * antipsych.1 + 0.13099133 * statin - 0 * fibrates - 0 * valproic - 0 * beta.blockers + 0.200257171 * thiazide - 0 * niacin - 0 * glucocorticoids - 0 * protease.inhib - 0 * nrti - 0.301418048 * ocp - 0 * depo - 0 * cyclosporine - 0.448824231 * tacrolimus + 0.002427009 * gfr - 0.133995682 * HTN - 0 * IHD - 0 * PVD - 0.191724052 * NEUROPATHY - 0.276870911 * OBESITY - 0.041494492 * HYPERLIPIDEMIA - 0 *(race=="Other") - 0.892833106 *(race=="White or Caucasian") + 0.036442945 * age + 0.095201316 *(fh.generations=="1") + 0.081555848 *(fh.generations=="2 or 3") + 0.054643321 * bmi + 0.106095371 *(gender=="Male") - 0 * tc + 0.000712335 * sbp + 0.004197089 * dbp. The probability of elevated HbA1c (>=5.7%) = exp(linear predictor)/(1+exp(linear predictor)). | |

| **Supplemental Table 7.** Coefficients for approximation model from development cohort (EHR) and approximation model recalibrated in 2017-2020 (pre-pandemic) NHANES. | | | |
| --- | --- | --- | --- |
|  | **Approximation**  **(Coefficient [S.E.])** | **Logistic recalibration of approximation model in NHANES without fasting subsample weights**  **(Coefficient [S.E.])** | **Logistic recalibration of approximation model in NHANES with normalized fasting subsample weights**  **(Coefficient [S.E.])** |
| N | 22635 | 2348 | 2348 |
| Intercept | -6.3471 [0.3633] | -6.7864 [1.4687] | -6.8627 [1.6919] |
| Fasting blood glucose (mg/dL) | 0.0071 [0.0030] | 0.0078 [0.0126] | 0.0078 [0.0146] |
| Fasting blood glucose (mg/dL)' | 0.0320 [0.0033] | 0.0351 [0.0143] | 0.0353 [0.0164] |
| Smoking Status=Former Smoker | -0.3691 [0.0514] | -0.4050 [0.1629] | -0.4073 [0.1748] |
| Smoking Status=Never Smoker | -0.4212 [0.0446] | -0.4623 [0.1352] | -0.4648 [0.1521] |
| Non-HDL cholesterol (mg/dL) | 0.0163 [0.0025] | 0.0179 [0.0069] | 0.0180 [0.0074] |
| Non-HDL cholesterol (mg/dL)' | 0.0041 [0.0027] | 0.0045 [0.0077] | 0.0045 [0.0082] |
| BMI (kg/m²) | 0.0627 [0.0068] | 0.0688 [0.0238] | 0.0692 [0.0266] |
| BMI (kg/m²)' | -0.0157 [0.0081] | -0.0172 [0.0278] | -0.0173 [0.0306] |
| eGFR (mL/min/1.73 m²) | 0.0102 [0.0011] | 0.0112 [0.0056] | 0.0113 [0.0065] |
| eGFR (mL/min/1.73 m²)' | -0.0098 [0.0014] | -0.0107 [0.0077] | -0.0108 [0.0088] |
| Race=Other | -0.1033 [0.0688] | -0.1134 [0.1412] | -0.1140 [0.1973] |
| Race=White or Caucasian | -1.0373 [0.0404] | -1.1384 [0.1433] | -1.1447 [0.1795] |
| Age (years) | 0.0595 [0.0035] | 0.0653 [0.0117] | 0.0656 [0.0130] |
| Age (years)' | -0.0213 [0.0034] | -0.0233 [0.0132] | -0.0235 [0.0147] |
| Total cholesterol (mg/dL) | -0.0126 [0.0022] | -0.0138 [0.0064] | -0.0139 [0.0072] |
| Total cholesterol (mg/dL)' | -0.0035 [0.0025] | -0.0039 [0.0074] | -0.0039 [0.0080] |
| Continuous variables were fit using restricted cubic splines with 3 knots.  The ' symbol indicates a spline term. | | | |

| **Supplemental Table 8**. Approximation model from development cohort (EHR) for predicting the probability of glycated hemoglobin ≥5.7%. |
| --- |
| N = 22635 |
| linear predictor = -6.3470619+0.0070710681* redo.glu+0.000027645977*pmax(redo.glu-74,0)^3-0.000049471748*pmax(redo.glu-89,0)^3+0.000021825771*pmax(redo.glu-108,0)^3-0.36910535*(smoking.status=="Former Smoker")-0.42123849*(smoking.status=="Never Smoker")+0.016324462* non.hdl+0.00000046090905*pmax(non.hdl-92,0)^3-0.00000083318174*pmax(non.hdl-134,0)^3+0.00000037227269*pmax(non.hdl-186,0)^3+0.010248498* gfr-0.0000015909845*pmax(gfr-50.516046,0)^3+0.0000037623329*pmax(gfr-95.739809,0)^3-0.0000021713485*pmax(gfr-128.87604,0)^3-0.10334226*(race=="Other")-1.0373426*(race=="White or Caucasian")+0.0594932* age-0.000012911351*pmax(age-29.276934,0)^3+0.000025891879*pmax(age-49.620808,0)^3-0.000012980528*pmax(age-69.856263,0)^3+0.062692301* bmi-0.00004455835*pmax(bmi-22.305816,0)^3+0.000072102741*pmax(bmi-29.475615,0)^3-0.000027544391*pmax(bmi-41.074144,0)^3-0.012601627* tc-0.00000035965146*pmax(tc-139,0)^3+0.000000659361*pmax(tc-184,0)^3-0.00000029970955*pmax(tc-238,0)^3 |
| The probability of elevated HbA1c (>=5.7%) = exp(linear predictor)/(1+exp(linear predictor)). |

| **Supplemental Table 9**. Approximation model recalibrated in NHANES 2017-2020 (pre-pandemic) for predicting the probability of glycated hemoglobin ≥5.7%. |
| --- |
| N = 2348 |
| linear predictor = -6.7863754+0.0077596263* redo.glu+0.000051879868*pmax(redo.glu-89,0)^3-0.000089925104*pmax(redo.glu-100,0)^3+0.000038045236*pmax(redo.glu-115,0)^3-0.40504766*(smoking.status=="Former Smoker")-0.46225736*(smoking.status=="Never Smoker")+0.017914086* non.hdl+0.00000045599107*pmax(non.hdl-85,0)^3-0.00000080612707*pmax(non.hdl-128,0)^3+0.000000350136*pmax(non.hdl-184,0)^3+0.068797079* bmi-0.00006030629*pmax(bmi-21.3,0)^3+0.000097997722*pmax(bmi-27.8,0)^3-0.000037691432*pmax(bmi-38.2,0)^3+0.011246465* gfr-0.000003676402*pmax(gfr-69,0)^3+0.0000082719046*pmax(gfr-99,0)^3-0.0000045955026*pmax(gfr-123,0)^3-0.1134054*(race=="Other")-1.1383557*(race=="White or Caucasian")+0.06528646* age-0.000010561886*pmax(age-25,0)^3+0.00001909264*pmax(age-46,0)^3-0.000008530754*pmax(age-72,0)^3-0.013828733* tc-0.00000038681918*pmax(tc-139,0)^3+0.00000069074853*pmax(tc-183,0)^3-0.00000030392935*pmax(tc-239,0)^3 |
| The probability of elevated HbA1c (>=5.7%) = exp(linear predictor)/(1+exp(linear predictor)). |

| **Supplemental Table 10**. Approximation model recalibrated in NHANES 2017-2020 (pre-pandemic) with normalized fasting subsample weights for predicting the probability of glycated hemoglobin ≥5.7%. |
| --- |
| N = 2348 |
| linear predictor = -6.8627361+0.0078026359* redo.glu+0.000052167424*pmax(redo.glu-89,0)^3-0.000090423535*pmax(redo.glu-100,0)^3+0.000038256111*pmax(redo.glu-115,0)^3-0.40729273*(smoking.status=="Former Smoker")-0.46481953*(smoking.status=="Never Smoker")+0.018013379* non.hdl+0.00000045851851*pmax(non.hdl-85,0)^3-0.00000081059522*pmax(non.hdl-128,0)^3+0.00000035207671*pmax(non.hdl-184,0)^3+0.069178403* bmi-0.000060640552*pmax(bmi-21.3,0)^3+0.000098540898*pmax(bmi-27.8,0)^3-0.000037900345*pmax(bmi-38.2,0)^3+0.011308801* gfr-0.0000036967794*pmax(gfr-69,0)^3+0.0000083177536*pmax(gfr-99,0)^3-0.0000046209742*pmax(gfr-123,0)^3-0.11403398*(race=="Other")-1.1446653*(race=="White or Caucasian")+0.065648325* age-0.000010620428*pmax(age-25,0)^3+0.000019198465*pmax(age-46,0)^3-0.0000085780378*pmax(age-72,0)^3-0.013905382* tc-0.00000038896322*pmax(tc-139,0)^3+0.00000069457717*pmax(tc-183,0)^3-0.00000030561396*pmax(tc-239,0)^3 |
| The probability of elevated HbA1c (>=5.7%) = exp(linear predictor)/(1+exp(linear predictor)). |

**Supplemental Figure** **8**. Mean absolute prediction error (MAPE) plot for the LASSO model developed using electronic health record data. The average mean absolute prediction error across all individuals, that is, the average mean absolute difference between individuals’ original and 2,000 bootstrap LASSO model predictions is 0.011.


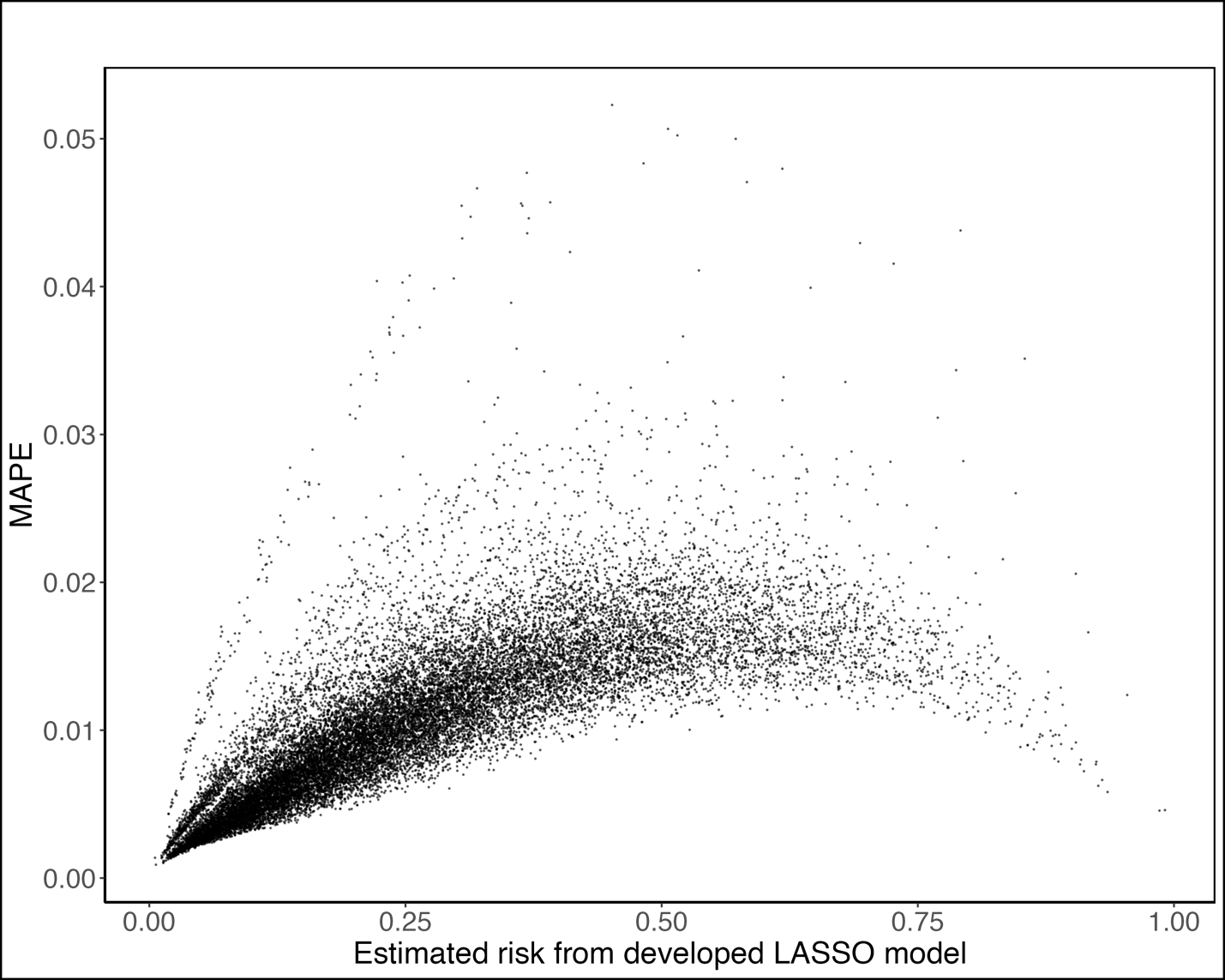


**Supplemental Figure** **9**. Prediction instability plot for the LASSO model developed using electronic health record data. The 2,000 bootstrap model predicted values (y-axis) for each individual is scattered against their original predicted value (x-axis). The dashed line illustrates the 95% range of predictions from the bootstrap models.


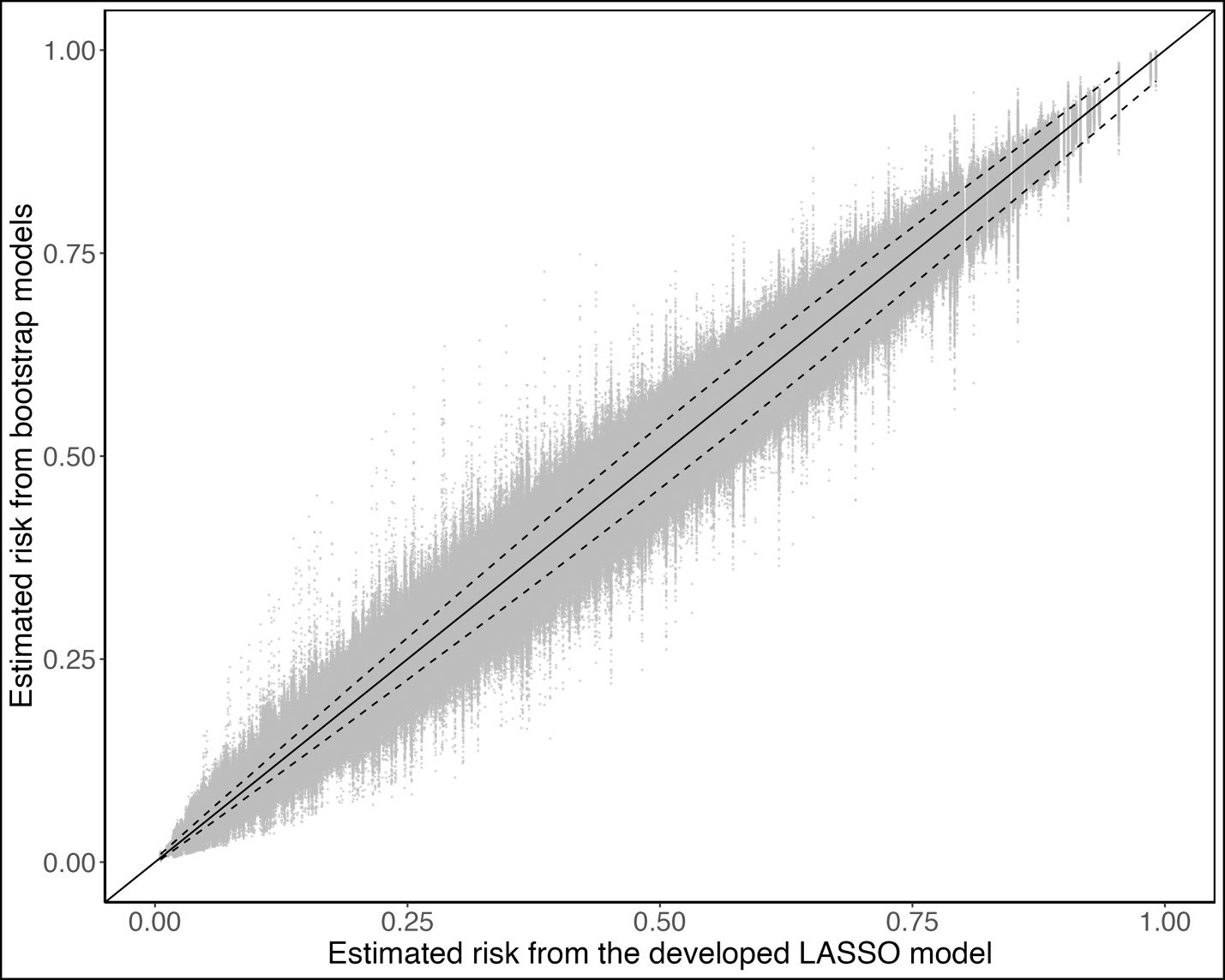


**Supplemental Figure** **10**. Calibration instability plot for the LASSO model developed using electronic health record data. Plot displays smoothed calibration curves for 2,000 bootstrap models calibrated to the original (development) electronic health record data.


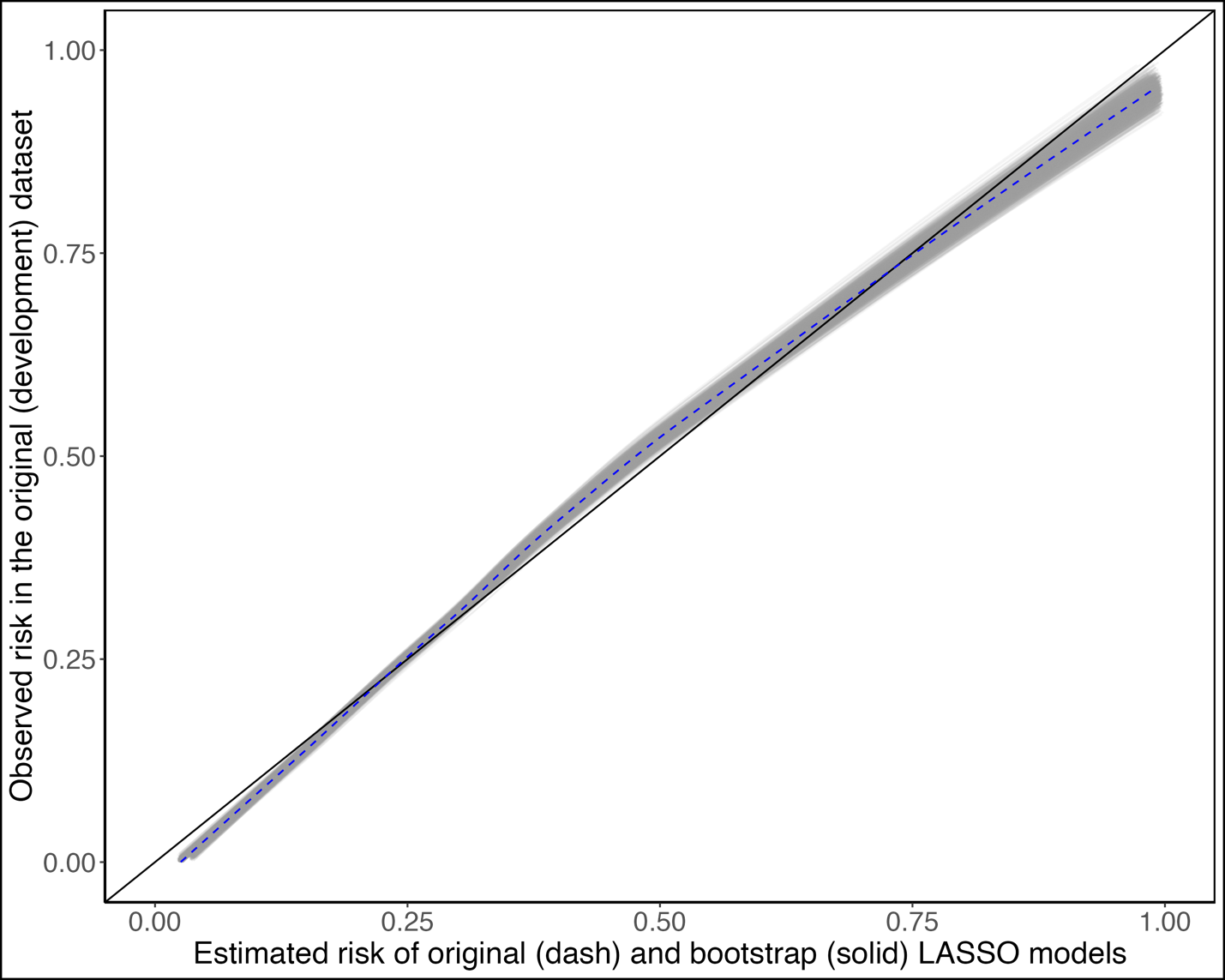


**Supplemental Figure** **11**. Mean absolute prediction error (MAPE) plot for the approximation model developed using electronic health record data. The average mean absolute prediction error across all individuals, that is, the average mean absolute difference between individuals’ original and 2,000 bootstrap approximation model predictions is 0.018.


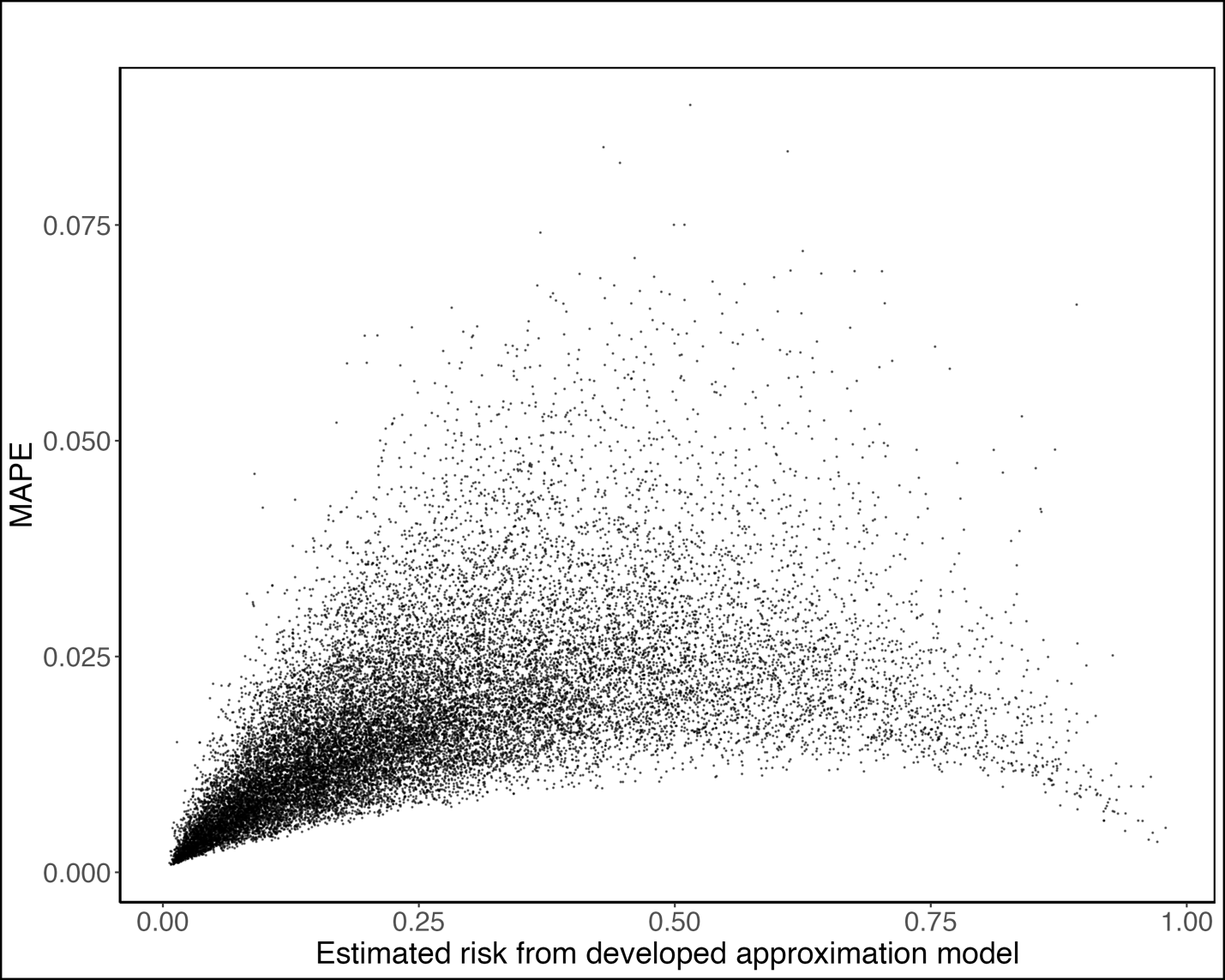


**Supplemental Figure** **12**. Prediction instability plot for the approximation model developed using electronic health record data. The 2,000 bootstrap model predicted values (y-axis) for each individual is scattered against their original predicted value (x-axis). The dashed line illustrates the 95% range of predictions from the bootstrap models.


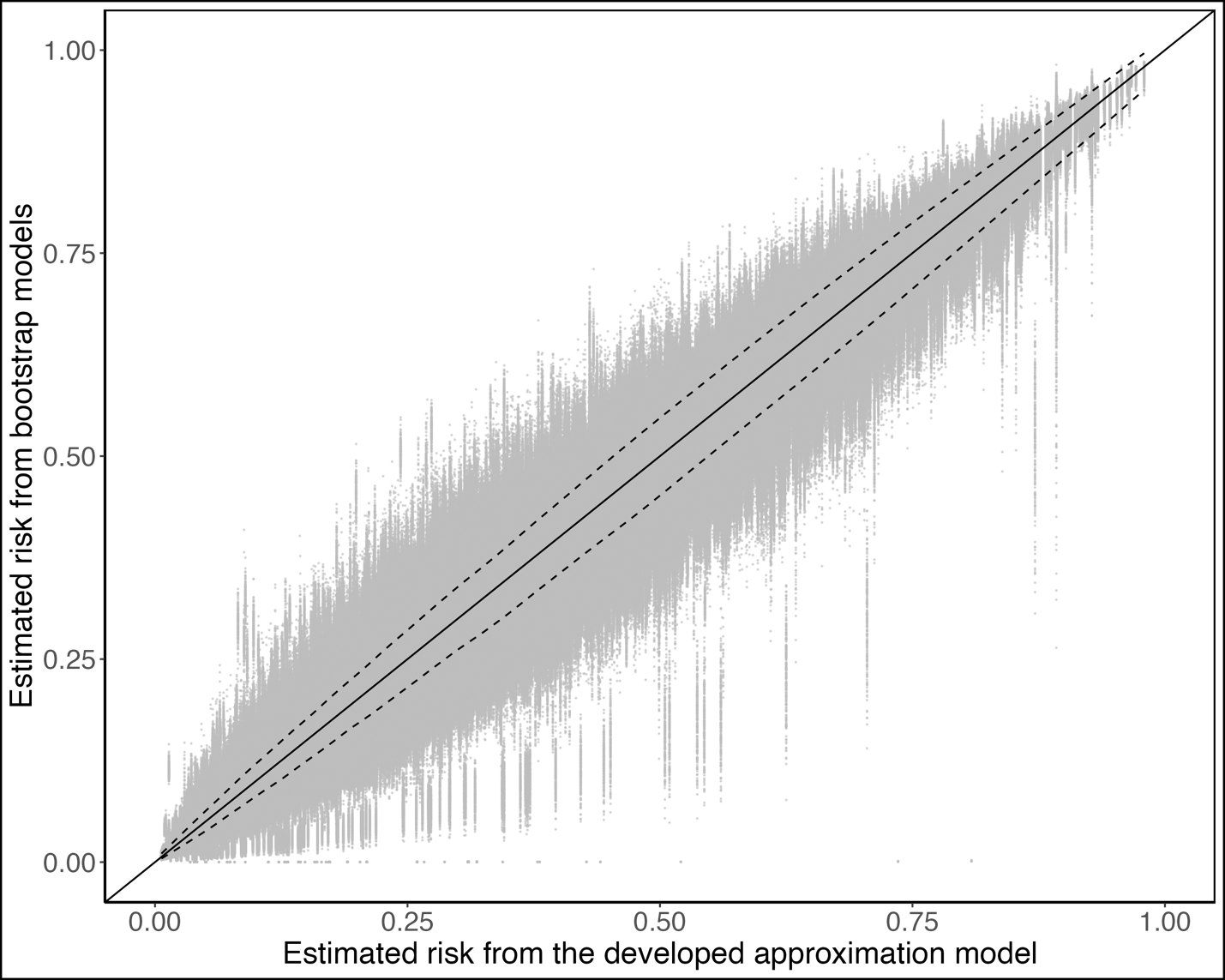


**Supplemental Figure** **13**. Calibration instability plot for the approximation model developed using electronic health record data. Plot displays smoothed calibration curves for 2,000 bootstrap models calibrated to the original (development) electronic health record data.


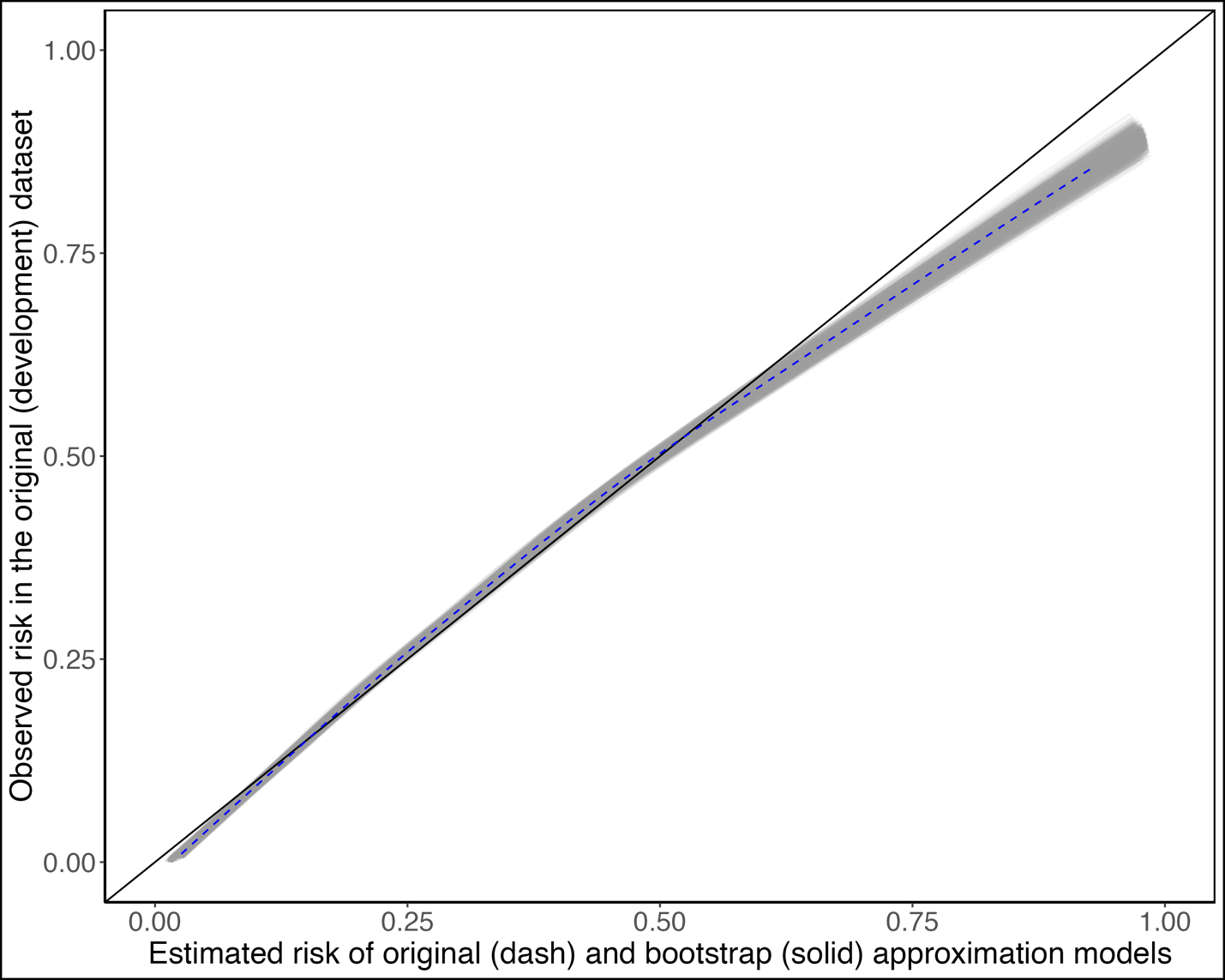

Supplement: Supplementary file 2 — Supplementary Material 2. [file 12911_2024_2803_MOESM2_ESM.docx]
